# Cutaneous Lyme borreliosis: Guideline of the German Dermatology Society

**DOI:** 10.3205/000255

**Published:** 2017-09-05

**Authors:** Heidelore Hofmann, Volker Fingerle, Klaus-Peter Hunfeld, Hans-Iko Huppertz, Andreas Krause, Sebastian Rauer, Bernhard Ruf, Consensus group

**Affiliations:** 1 Klinik für Dermatologie und Allergologie der TU München, München, Germany; 2 Bayerisches Landesamt für Gesundheit und Lebensmittelsicherheit (LGL) Oberschleißheim, Germany; 3 Zentralinstitut für Labormedizin, Mikrobiologie & Krankenhaushygiene, Krankenhaus Nordwest, Frankfurt, Germany; 4 Professor-Hess-Kinderklinik Klinikum Bremen-Mitte, Bremen, Germany; 5 Immanuel Krankenhaus Berlin, Berlin, Germany; 6 Neurologische Universitätsklinik, Freiburg, Germany; 7 Klinik für Infektiologie Klinik St Georg, Leipzig, Germany

## Abstract

This guideline of the German Dermatology Society primarily focuses on the diagnosis and treatment of cutaneous manifestations of Lyme borreliosis. It has received consensus from 22 German medical societies and 2 German patient organisations. It is the first part of an AWMF (Arbeitsgemeinschaft der Wissenschaftlichen Medizinischen Fachgesellschaften e.V.) interdisciplinary guideline: “Lyme Borreliosis – Diagnosis and Treatment, development stage S3”.

The guideline is directed at physicians in private practices and clinics who treat Lyme borreliosis. Objectives of this guideline are recommendations for confirming a clinical diagnosis, recommendations for a stage-related laboratory diagnosis (serological detection of IgM and IgG Borrelia antibodies using the 2-tiered ELISA/immunoblot process, sensible use of molecular diagnostic and culture procedures) and recommendations for the treatment of the localised, early-stage infection (erythema migrans, erythema chronicum migrans, and borrelial lymphocytoma), the disseminated early-stage infection (multiple erythemata migrantia, flu-like symptoms) and treatment of the late-stage infection (acrodermatitis chronica atrophicans with and without neurological manifestations). In addition, an information sheet for patients containing recommendations for the prevention of Lyme borreliosis is attached to the guideline.

## Zusammenfassung

Diese Leitlinie der Deutschen Dermatologischen Gesellschaft bezieht sich primär auf die Diagnostik und Therapie von kutanen Manifestationen der Lyme-Borreliose. Sie ist Teil 1 der geplanten interdisziplinären AWMF Gesamtleitlinie „Lyme-Borreliose – Diagnostik und Therapie Entwicklungsstufe S3“.

Sie wurde interdisziplinär von 22 deutschen Fachgesellschaften und 2 deutschen Patientenorganisationen konsentiert und richtet sich an Ärzte in Praxis und Klinik, die mit der Behandlung der Lyme-Borreliose befasst sind.

Ziel der Leitlinie ist es, Empfehlungen zur Absicherung der klinischen Diagnosen, Empfehlungen zur stadiengerechten Labordiagnostik (serologischer Nachweis von IgM- und IgG-Borrelienantikörpern mit dem 2-Stufenverfahren ELISA/Immunoblot sowie der sinnvolle Einsatz molekulardiagnostischer und kultureller Verfahren) und Empfehlungen zur Therapie der lokalisierten Frühmanifestationen (Erythema migrans, Erythema chronicum migrans und Borrelienlymphozytom), zur Therapie der disseminierten Frühmanifestationen (Multiple Erythemata migrantia und/oder grippeartige Symptomatik nach Zeckenstich) und zur Therapie der Spätmanifestationen (Acrodermatitis chronica atrophicans mit und ohne neurologische Manifestationen) zu geben. Außerdem werden Empfehlungen für Patienten zur Prävention der Lyme-Borreliose und zur Nachbeobachtung eines Zeckenstiches formuliert.

## Preamble

This guideline primarily focuses on the diagnosis and treatment of cutaneous manifestations of Lyme borreliosis. It is the first part of the scheduled interdisciplinary guideline: *“Lyme Borreliosis – Diagnosis and Treatment, No. 013-080, Development Stage S3*”. 

This part has already received consensus from 22 medical societies and 2 patient organisations. The German Cochrane Centre, Freiburg (Cochrane Germany) is currently conducting systematic review and assessment of the literature to develop this guideline to stage 3. 

The interdisciplinary guideline group is currently preparing part 2 “Neuroborreliosis” which will be followed by part 3 “Lyme Arthritis, Lyme Carditis and Other Rare Manifestations”.

### Synonyms

Cutaneous borreliosis, cutaneous manifestations of Lyme borreliosis, skin borreliosis, cutaneous Lyme borreliosis, cutaneous Lyme disease

### Search terms

*Borrelia burgdorferi* infection, hard-bodied tick borreliosis, Lyme disease, cutaneous Lyme borreliosis, erythema migrans disease, erythema migrans, erythema chronicum migrans, lymphadenosis cutis benigna, borrelial lymphocytoma, multiple erythemata migrantia, multiple erythema migrans, acrodermatitis chronica atrophicans.

ICD-10-No: A69.2, L90.4 

AWMF Register No 013-044 

## List of abbreviations

- ACA – Acrodermatitis chronica atrophicans
- BL – Borrelial lymphocytoma
- EM – Erythema migrans
- ECM – Erythema chronicum migrans
- i.v. – Intravenous
- BW – Body weight
- LB – Lyme borreliosis
- LTT – Lymphocyte transformation test
- MEM – Multiple Erythemata migrantia
- MiQ – Quality standards in microbiological-infectiological diagnostics
- NAT – Nucleic acid amplification techniques
- NSAID – Nonsteroidal anti-inflammatory drugs
- PCR – Polymerase chain reaction
- p.o. – Per os
- PPI – Proton pump inhibitor
- RCT – Randomised controlled trial
- SNRI – Serotonin-norepinephrine reuptake inhibitor

## 1 Introduction

The infectious disease most frequently transmitted by ticks in Europe is Lyme borreliosis. The Borrelia are transferred to the skin during the blood sucking process of the hard-bodied tick *Ixodes ricinus*. There the Borrelia are either killed off by the (unspecific, innate) immune system, or a localised infection occurs which leads to illness in only a small percentage of those infected. Most often there is an inflammation of the skin, typically in the form of an erythema migrans or, seldom, as borrelial lymphocytoma. In the course of the infection the Borrelia can disseminate and attack various organs. They primarily affect the skin, joints and nervous system. Acrodermatitis chronica atrophicans can develop as a chronic or late-form of skin manifestation. 

### 1.1 Target group 

This guideline is directed at physicians in private practices and clinics who treat Lyme borreliosis. 

### 1.2 Objectives of this guideline

- Recommendations for confirming a clinical diagnosis
- Recommendations for a stage-related laboratory diagnosis: serological detection of IgM and IgG Borrelia antibodies using the 2-tiered ELISA/immunoblot process; sensible use of molecular-diagnostic and culture procedures
- Treatment of the localised, early-stage infection (erythema migrans, erythema chronicum migrans and borrelial lymphocytoma)
- Treatment of the disseminated early-stage infection (multiple erythemata migrantia, flu-like symptoms)
- Treatment of the late-stage infection (acrodermatitis chronica without neurological manifestations)
- Treatment of the late-stage infection (acrodermatitis chronica with neurological manifestations)
- Prevention of Lyme borreliosis
  Recommendations for observing the area around the tick bite
  Information sheet for patients (Annex 1 in Attachment 1 )

### 1.3 Participating medical societies 

**Steering group**

- Responsible:
  Prof. Dr. med. Heidelore Hofmann – coordinator
  German Dermatology Society (DDG)
- Prof. Dr. med. Sebastian Rauer – coordinator, deputy Dr. Stephan Kastenbauer
  German Society of Neurology (DGN)
- Dr. med. Volker Fingerle
  German Society for Hygiene and Microbiology (DGHM)
- Prof. Dr. med. Klaus-Peter Hunfeld
  The German United Society of Clinical Chemistry and Laboratory Medicine (DGKL) and INSTAND e.V.
- Prof. Dr. med. Hans-Iko Huppertz
  German Society of Paediatrics and Adolescent Medicine (DGKJ) and German Society of Paediatric Infectology (DGPI)
- Prof. Dr. med. Andreas Krause
  German Society of Rheumatology (DGRh)
- Prof. Dr. med. Bernhard Ruf
  German Society of Infectious Diseases (DGI)

**Consensus group **

- Prof. Dr. med. Elisabeth Aberer
  Austrian Society of Dermatology and Venerology (ÖGDV)
- Prof. Dr. med. Karl Bechter
  The German Association of Psychiatry, Psychotherapy and Psychosomatics (DGPPN)
- Prof. Dr. med. Michael H. Freitag
  German College of General Practitioners and Family Physicians (DEGAM)
- PD Dr. med. Gudrun Goßrau
  German Pain Society (DGSS)
- Prof. Dr. med. Gerd Gross
  Paul Ehrlich Society for Chemotherapy (PEG)
- Prof. Dr. med. Rainer Müller
  German Society of Oto-Rhino-Laryngology, Head and Neck Surgery (DGHNOKHC)
- Dr. med. Kurt Müller / PD Dr. med. Walter Berghoff
  German Borreliosis Society (DBG)
- Prof. Dr. med. Mathias Pauschinger
  German Society of Cardiology and Cardiovascular Research (DGK)
- Prof. Dr. med. Monika A. Rieger
  German Society for Occupational and Environmental Medicine (DGAUM)
- PD Dr. med. Rainer Schäfert
  German Society of Psychosomatic Medicine and Medical Psychotherapy (DGPM) and the German College of Psychosomatic Medicine (DKPM)
- Prof. Dr. med. Stephan Thurau
  German Ophthalmological Society (DOG)
- Prof. Dr. rer. nat. Reinhard Wallich
  German Society for Immunology (DGI)
- Dr. Hendrik Wilking
  Robert Koch Institute (RKI)

**Patient supporting groups**

- Ursula Dahlem
  Action Alliance Against Tick-Borne Infections Germany (OnLyme-Aktion)
- Ute Fischer / Karin Friz
  Borreliosis and FSME Association Germany (BFBD)

**Moderation**

- Prof. Dr. med. Ina B. Kopp
  AWMF Institute for Medical Knowledge Management

### 1.4 Methods

This guideline is based on an update of AWMF Guideline No. 013-044 “Cutaneous Manifestations of Lyme Borreliosis”, development stage S1, which was created by a committee of experts in 2009. 

The guideline was created in accordance with the methodological requirements of the Association of the Scientific Medical Societies in Germany (AWMF) for developing and further developing diagnosis and treatment guidelines. It is an S2k guideline in accordance with the AWMF’s three-stage concept. The composition of the guideline group was interdisciplinary (IDA) and the appointed mandate holders of the expert medical societies were informed of the scheduled update on 11/2/2014. 

Uniform formulations are used in order to standardise the recommendations of the guideline. 

The following gradations shall apply here:

- **Strong recommendation: “shall” **
- **Recommendation: “should” **
- **Open recommendation: “may be considered” **
- **Recommendation against an intervention: “should not” **
- **Strong recommendation against an intervention: “shall not” **

## 2 Microbiology of the pathogen

In Europe, **5 human-pathogenic genospecies from the Borrelia burgdorferi sensu lato complex have so far been isolated**: *B. afzelii* is the most frequent, followed by *B. garinii*, *B. bavariensis*, *B. burgdorferi* sensu stricto and *B. spielmanii* [1], [2], [3], [4].

The human pathogenicity is still unclear for *B. valaisiana*, *B. lusitaniae* and *B. bissettii*. All of the species that have been ascertained to be human-pathogenic are found in Europe. Only B. burgdorferi sensu stricto is present in the USA, and all of the species are present in Asia except for *B. burgdorferi* sensu stricto. The various genospecies of the *Borrelia burgdorferi* sensu lato complex are genetically very heterogenic [5] and exhibit an organotropism in human infections. Erythema migrans is triggered by all 5 genospecies. Almost only *B. afzelii* is detected with acrodermatitis chronica atrophicans, *B. garinii* and *B. bavariensis* are often present in neurological manifestations, and *B. burgdorferi* sensu stricto mainly affects the joints [6]. *B. spielmanii* has so far only been isolated from erythema migrans [2], [7]. 

## 3 Epidemiology

Lyme borreliosis mainly exists between the 40^th^ and 60^th^ parallels of the northern hemisphere in line with the presence of its vectors. Few relevant epidemiological investigations have been conducted in Europe. A population-based study in southern Sweden reveals an incidence of 69 per 100,000 inhabitants [8]. In a prospective, population-based study of the region around Würzburg over a 12 month period, 313 cases of Lyme borreliosis were reported, which corresponds to an incidence of 111 per 100,000 inhabitants [9]. In terms of early manifestations, a localised erythema migrans was diagnosed in 89% of the cases and a disseminated erythema migrans in a further 3% of cases. Borrelial lymphocytoma was established in 2% of cases, early-stage neuroborreliosis in 3%, and carditis in <1%. In terms of late-stage forms of the disease, Lyme arthritis appeared in 5% of patients and acrodermatitis chronica atrophicans in 1%. No chronic neuroborreliosis was detected. 

Currently nine states in Germany have an obligation to report acute manifestations of Lyme borreliosis (see Annex 4 in Attachment 1). Epidemiological data obtained through this partial obligation to report are only based on the clearly diagnosable manifestations, such as erythema migrans, acute neuroborreliosis and acute Lyme arthritis. Thus, it can be assumed that the rate of incidence is considerably underreported [10], [11]. Secondary data analyses of health insurance data based on the ICD 10 coding A 69.2 (G) result in much higher rates of incidence [12]. 

Therefore, it can be concluded that the epidemiological data currently available is not sufficient for a definitive clarification. Data published up until now in Germany indicates the incidence of Lyme borreliosis to be somewhere between 60,000 to >200,000 cases per year. 

**Figure 1:**
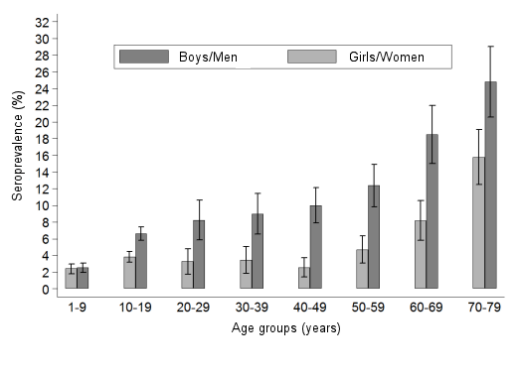
Seroprevalence of *B. burgdorferi* antibodies in Germany. KIGGS and DEGS studies [13]

In a major nation-wide seroprevalence study of children (KIGGS) and adults (DEGGS) it was shown that the percentage of Borrelia-specific antibodies in serum increases with increasing age of the population and already has an incidence rate of 7% in the group of 14 to 17 year olds. In adults, this percentage of Borrelia antibodies is even higher. In the group of 70 to 79 year olds, 24.5% of men and 16.4% of women are seropositive (Figure 1 (Fig. 1)) [13]. 

A prospective investigation of the incidence of Lyme borreliosis in Finland and southern Sweden (2008–2009) revealed that 78 (5%) of the 1,546 people bitten by a tick had a Borrelia burgdorferi infection. In 45 of the cases (3%) only a seroconversion occurred; 33 (2%) resulted in illness. Erythema migrans was diagnosed in 28 people, one person had borrelial lymphocytoma, two people had an acute case of neuroborreliosis and 2 had unspecified symptoms which were diagnosed as Lyme borreliosis [14]. 

## 4 Transmission routes

*B. burgdorferi* is transmitted to birds, mammals and humans from hard-bodied ticks of the *I. ricinus*/*I. persulcatus* spp. complex during the blood meal. In Europe this transmission is primarily from *I. ricinus*, in Asia from *I. persulcatus* and in the USA predominantly from *I. scapularis*. Ticks suck blood in the course of their cycle of development from larva to nymph to adult tick, and before they lay eggs. It is at this time that they can acquire and/or transmit Borrelia. Small rodents – particularly mice – and birds are the main reservoirs. Birds contribute to the geographical propagation of the infected ticks. In Germany, ticks are ubiquitously infected with Borrelia, however percentages can vary heavily from region to region, even between areas very close in proximity (e.g. 4–21% [15]). 

The successful transmission from tick to mammal is the result of a specific, highly complex vector-pathogen interaction. First the Borrelia are activated in the tick’s intestines. Then they travel to the salivary glands where they bind immunosuppressive salivary proteins to their surface [16]. Finally, they are secreted with the saliva in the bite wound where they are at least partially protected from the host’s immune system by immunomodulating substances from the tick’s saliva which probably allows them to reach a sufficiently high infection doses. A similar transmission through blood-sucking insects is therefore close to impossible due to the short blood sucking time (lack of vector competence in insects for *B. burgdorferi*). Xenobiotic tests reveal that it can take hours for the Borrelia to be transferred – depending on the species of Borrelia [17].

When there is an occupationally higher risk of tick bites, cases of Lyme borreliosis (occupational disease No. 3102, diseases transmitted from animals to humans) should be reported to the accident insurer by the attending physician or employer as a work-related illness as per Art. 202 of the Social Security Code VII (see Annex 4 in Attachment 1). 

## 5 Pathogenesis

The pathogenesis of the borrelial infection is primarily determined by two factors:

1. The evasion strategies of the pathogen [18], [19], [20].
2. The quality of the host’s immune response [21], [22], [23], [24], [25], [26], [27], [28].

Moreover, salivary proteins that are released in the course of the tick’s blood meal also show immunosuppressive effects [29], [30], [31], [32], [33], [34], [35], [36], [37].

Host-specific inflammatory reactions in the skin also influence the course of the infection [38], [39]. 

Some of the many strategies the Borrelia use to evade the host’s immune system include the ability to mask their cell surface with proteins/inhibitors from the tick or the host, and to modify their phenotype expression of cell surface proteins (outer surface protein: osp) according to their environment [40], [41], [42], [43].

Several Borrelia species form a resistance to complement-mediated lysis by binding the regulators of the complement cascade (factor H) to their surface [44], [45], [46], [47], [48]. By binding to plasminogens, Borrelia are capable of breaking down collagen, fibronectin and laminin [47], [49], [50], [51], [52] and disseminating in the skin. 

The innate immune system recognises the Borrelia mainly by their surface proteins (osp lipoproteins) [53], [54], [55], [56], [57]. This interaction leads to the activation of soluble factors, such as the complement system, as well as to the activation of target cells, like macrophages and dendritic cells, and to the induction of inflammatory cytokines [58], [59], [60], [61]. As the infection progresses, specific immune responses are generated, particularly the activation of T helper cells and B lymphocytes, and the production of Borrelia-specific antibodies [20], [62], [63], [64]. In reservoir hosts, like wild mice, the antibodies that form during an infection are able to prevent disease, however they are not able to eliminate the pathogen. In contrast, the antibodies that form in patients are often unable to prevent the disease. However, antibodies against certain Borrelia antigens have also been shown to protect against subsequent infection in humans (see vaccines). 

There is no permanent immunity in humans after wild-type infection. Thus reinfection can occur. 

## 6 Clinical manifestations of Lyme borreliosis

Lyme borreliosis is an inflammatory multi-organ disease. It manifests itself initially as a localised infection of the skin called erythema migrans. Because of its light symptoms, this early-stage inflammation of the skin can be overlooked or not even be visible. The Borrelia can spread haematogenically which is recognised clinically by flu-like symptoms or disseminated erythemas of the skin. As the disease progresses, manifestations can appear in other organs, with the nervous system and the joints primarily affected. The disease progresses very differently depending on the individual. Therefore, it doesn’t make sense to classify the disease into stages. A distinction between early and late manifestations is preferable since the clinical picture determines both the diagnosis and the treatment (Table 1 (Tab. 1)). European studies show that Lyme borreliosis manifests itself as a skin disease in 80–90% of patients and in other organs in around 10–20% of patients [8], [9], [10], [14], [65], [66].

### 6.1 Localised cutaneous early-stage infection 

#### 6.1.1 Erythema migrans 

**Figure 2:**
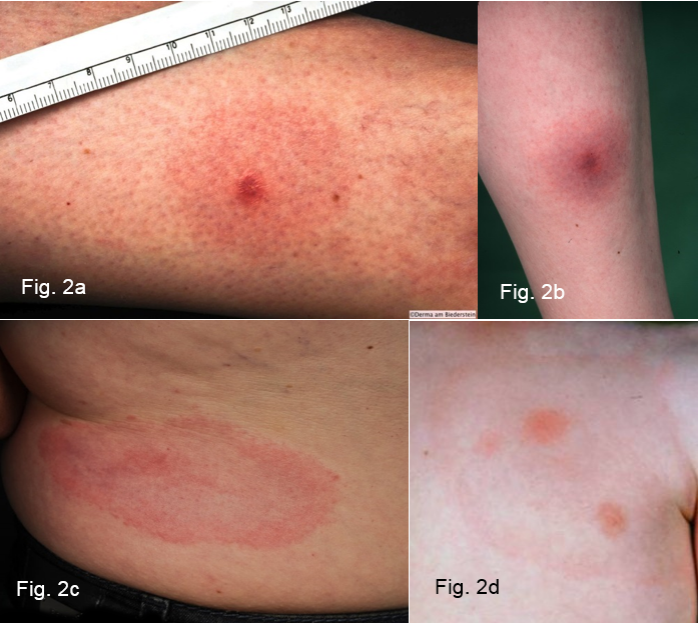
Clinical variations of erythema migrans Fig. 2a: Initial seronegative erythema migrans 1 week after tick bite DD reaction to insect bite; diameter 4.9 cm, progression under observation Fig. 2b: Seronegative erythema migrans, elevation of IgM antibodies occured 5 days after start of treatment Fig. 2c: Typical marginated migrating erythema migrans Fig. 2d: Typical marginated erythema migrans with fresh areas of inflammation within the ring

The skin around the infectious tick bite can become infected anywhere from 3 to 30 days after the tick bite occurs [67]. The extent and duration of the rash varies considerably between individuals. If the diameter of the erythema is more than 5 cm, a diagnosis of erythema migrans can be made (Figure 2a and b (Fig. 2)) [68].

The clinical picture of a typical **erythema migrans** is a marginated erythema that centrifugally spreads out around the tick bite (Figure 2c and d (Fig. 2)). 

**Features of a typical solitary erythema migrans **

- Free time interval between the tick bite and start of the erythema that is typically 3 days to several weeks. (Consensus: 18/20)
- Increasing centrifugal spreading of the erythema (crescendo reaction). (Consensus: 17/20)
- Marginated, non-raised erythema that is at least 5 cm in diameter. (Strong consensus: 20/20)
- A visible puncture site in the centre of the erythema. (Strong consensus: 20/20)

#### 6.1.2 Variability of the erythema migrans (atypical erythema migrans)

**Figure 3:**
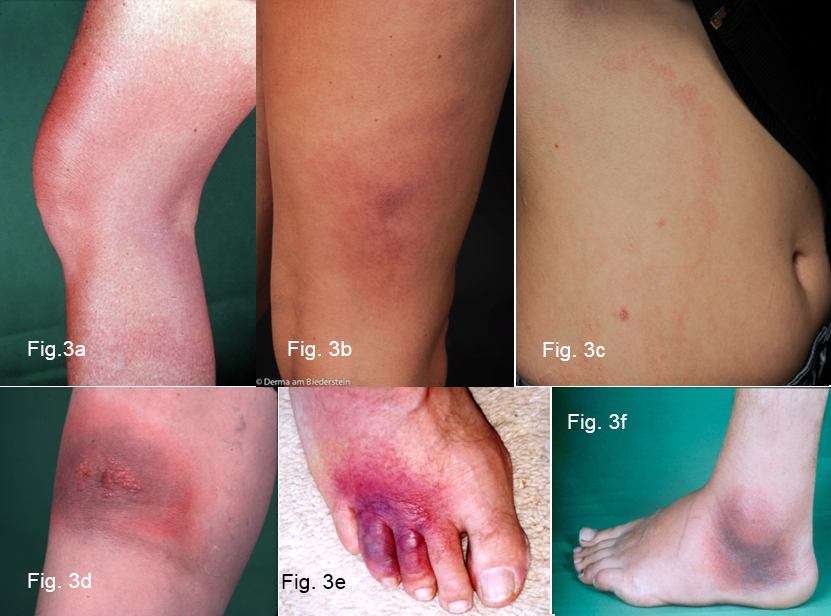
Variability of the erythema migrans Fig. 3a: Flaming red erythema chronicum migrans with radiculitis on the left leg, DD erysipelas Fig. 3b: Blotchy purple erythema chronicum migrans on the upper thigh for 3 months Fig. 3c: Large light-red arch-shaped erythema chronicum migrans on the abdomen Fig. 3d: Centrally vesicular erythema migrans Fig. 3e: Haemorrhagic bullous erythema migrans on the foot Fig. 3f: Purple, haemorrhagic, non-migrating erythema chronicum migrans on the outer ankle with joint swelling

Very often the initial skin infection cannot be definitively diagnosed clinically. Borrelia have been identified in homogenously red and non-migrating erythemas, spotty and infiltrated erythemas (Figure 3b (Fig. 3)), erysipelas-like flaming red erythemas (Figure 3a (Fig. 3)) and in centrally vesicular erythemas (Figure 3d (Fig. 3)) [69], [70]. The inflammation can completely disappear in the middle and fade to such as extent that the erythema is only visible around the edges – in the area of the migrating Borrelia – when heat is applied (Figure 3c (Fig. 3)). The erythema can also be haemorrhagic, particularly on the lower extremities (Figure 3 e and f (Fig. 3)). The centre can turn a dark purple colour (Figure 3f (Fig. 3)). The edge can be raised or urticarial. The former puncture site can be identified in the centre as a red papule (Figure 2a and b (Fig. 2)) [70], [71]. **Without antibiotic treatment** the Borrelia can persist for months or years in the skin and the erythema can slowly spread throughout the body. Often the red edge is the only evidence of the inflammatory reaction to the migrating Borrelia. If the erythema migrans persists for multiple weeks and months, it is referred to as **erythema chronicum migrans** ([66]: >4 weeks). In most cases (approx. 80%) serological detection of the IgG antibodies (sometimes even the IgM antibodies) is possible [72].

Erythema can disapear even without antibiotic treatment. Spontaneous healing is possible, however the Borrelia can persist even without a visible inflammatory reaction and, after a period of latency, this can lead to further organ manifestations. 

**Variability of the erythema migrans (atypical erythema migrans)**

- Non-migrating
- Not marginated
- Infiltrated instead of macular
- Centrally vesicular
- Haemorrhagic
- Irregular blotches
- Only visible when heat is applied to the skin
- No visible tick puncture site
  (Strong consensus 20/20)

**Concluding recommendation: **

Due to the extraordinary variability of the clinical presentation, atypical erythema migrans is difficult for dermatologically inexperienced physicians to diagnose. Therefore, patients with atypical erythema should be referred to a dermatologist. (Strong consensus 19/20)

#### 6.1.3 Borrelial lymphocytoma 

**Figure 4:**
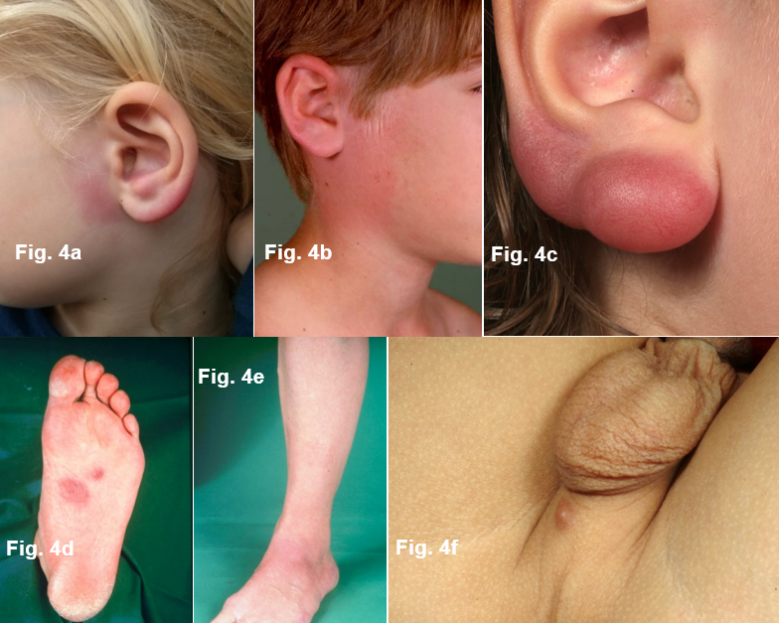
Borrelial lymphocytoma Fig. 4a: Borrelial lymphocytoma preauricular and on the left earlobe Fig. 4b: Borrelial lymphocytoma on the right auricle near a non-marginated erythema migrans Fig. 4c: Pronounced nodular borrelial lymphocytoma on the earlobe Fig. 4d/e: Borrelial lymphocytoma on the sole of the foot with erythema migrans on the lower leg, histologically initially misdiagnosed as a low-grade malignant B cell lymphoma Fig. 4f: Small perineal borrelial lymphocytoma

Pseudolymphoma (cutaneous lymphoid hyperplasia) can occur in the early stages at the puncture site or in the migrating erythema migrans (Figure 4b (Fig. 4)). Mostly it is solitary, in rare cases it is also disseminated. Borrelial lymphocytoma occurs more frequently in children than in adults (7% in children and only 2% in adults with Lyme borreliosis, [8]). The favoured sites in children are the earlobes (Figure 4a and 4c (Fig. 4)), nipples and genital-anal area (Figure 4f (Fig. 4)) [73]. The disease was first described as lymphadenosis cutis benigna by Bäferstedt in 1944. *B. burgdorferi* s.l. can be detected in the pseudolymphomas [74]. Mostly it is a case of *B. afzelii* [75]. From a histological perspective, there are mixed B and T lymphocytic infiltrates. However purely B cell infiltrates can also occur which are difficult to differentiate from low-grade B cell lymphoma (Figure 4d and e (Fig. 4)) [70]. Borrelial lymphocytoma can also occur in the late stages as part of an acrodermatitis chronic atrophicans [73]. 

In the case of borrelial lymphocytoma, a substantial increase in the number of IgG antibodies can be detected in the serum regardless of the length of infection [65], [76]. In rare cases, multiple borrelial lymphocytomas can occur in the early disseminated stages or even in the late stages of the disease. In these cases, precise histological, immune-histochemical and molecular-genetic clarification is required in order to diagnostically differentiate them from malignant cutaneous lymphomas. 

**Significant features of borrelial lymphocytoma **

- Pseudolymphoma, mostly solitary, more frequent in children
- Localised, above all on the earlobes, nipples or in the genital area
- Purple subcutaneous nodules or plaque
- Histologically mostly mixed B and T lymphocytic infiltrates
  (Strong consensus: 19/20)

### 6.2 Disseminated cutaneous early manifestation 

Some of the patients experience haematogenous dissemination in the early stages of the disease which can be identified by flu-like symptoms such as a slight fever, arthralgia, myalgia, headaches, lymphadenopathy and multiple erythemata migrantia. This stage is very difficult to diagnose if no erythemas are visible, or cannot be identified due to an atypical morphology. 

#### Multiple erythemata migrantia (MEM)

**Figure 5:**
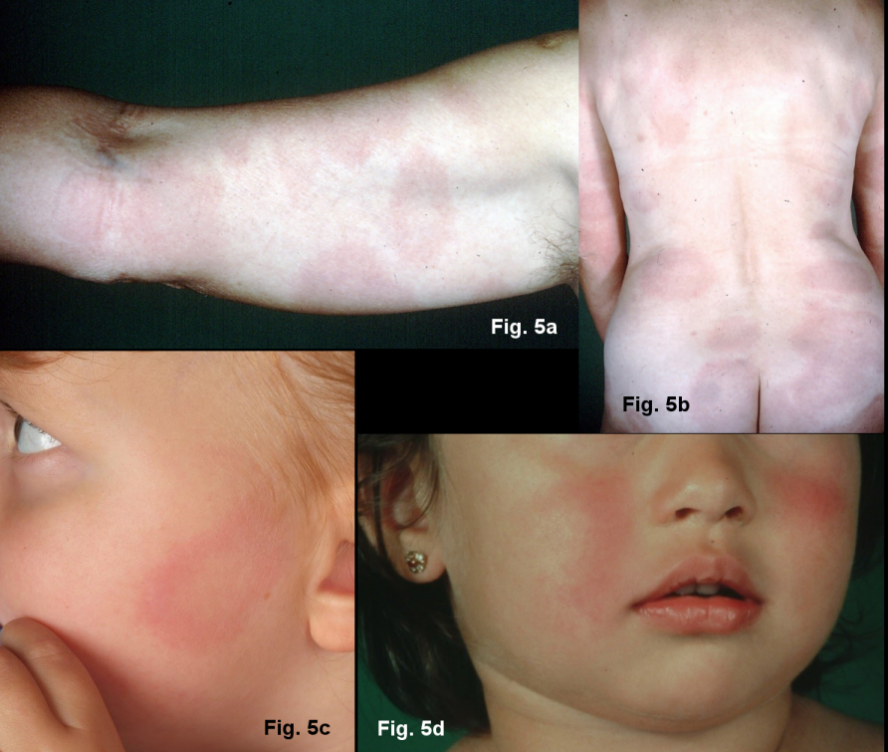
Multiple erythemata migrantia (MEM) Fig. 5a and 5b: Pronounced erythemata migrantia on the right arm, approx. 40 more on the torso and lower extremities Fig. 5c: Oval erythema on the left cheek, many similar erythemas on the torso and upper leg a sign of early disseminated borreliosis Fig. 5d: Symmetrical redness on the cheeks of a 5-year-old girl with multiple erythemata migrantia on her trunk and extremities, accompanied by flu-like symptoms

The haematogenous dissemination of the Borrelia in the skin is noticeable by the many sharply marginated, symptomless, oval erythemas of various sizes: multiple erythemata migrantia (Figure 5b and 5c (Fig. 5)) [69], [77], [78]. Children often experience symmetrical erythemas on their face, similar to fifth disease (parvovirus B 19 infection) (Figure 5a (Fig. 5)) [68], [70]. MEM can be associated with systemic symptoms and acute neurological symptoms [79]. The histological picture is initially atypical. The typical perivascular plasma-cellular infiltrates are not found until the advanced stage of the disease. There is usually a strong increase in IgM antibodies in the serum or the antibodies increase rapidly once treatment begins. There is usually an increase in IgG antibodies. Borrelia taken from skin lesions and, in rare cases, blood can be cultivated or their DNA can be detected using PCR [78], [80]. 

**Significant features of multiple erythemata migrantia**

- Symptomless, disseminated, round or oval redness on the skin (Strong consensus: 19/20)
  - Without epidermal changes
  - Ring-shaped or homogenous
  - Often symmetrical erythemas on the face of children (similar to fifth disease)
  - Persisting over days or weeks
  - Recurring at the same places
  - Possible association with systemic or acute neurological symptoms
    (Strong consensus: 19/20)

### 6.3 Cutaneous late manifestations

#### Acrodermatitis chronica atrophicans (ACA) 

The disease can manifest itself in various organs after varying periods of time, from months to years depending on the individual. A chronic skin infection mostly occurs in older people and more frequently in women [81]. Isolated cases have also been reported in children [82], [83], [84]. 

##### Oedematous infiltrative stage of ACA

**Figure 6:**
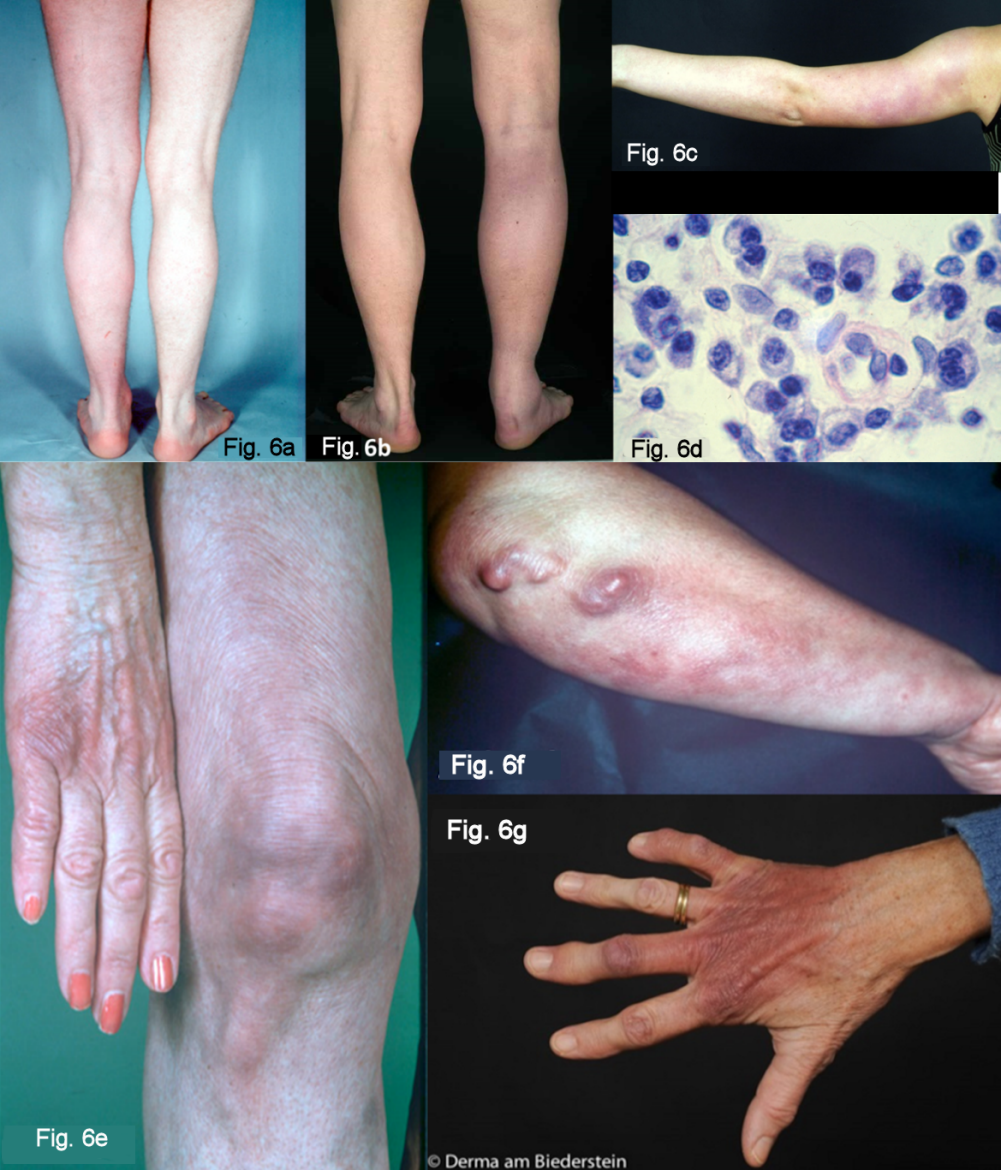
Late cutaneous manifestations Fig. 6a–d: Oedematous infiltrative stage of acrodermatitis chronica Fig. 6a: Acrodermatitis chronica. Homogenous reddening of the left leg without atrophy, persisting for one year Fig. 6b: Acrodermatitis chronica in the oedematous infiltrative stage. Hard swelling and purple colouring of the right leg with swelling of the Achilles tendon and swelling of the ankle joint Fig. 6c: Acrodermatitis in the oedematous infiltrative stage. Blotchy purple confluent erythemas on the left arm of a 15-year-old girl Fig. 6d: Typical perivascular plasma-cellular infiltrate in the case of acrodermatitis chronica Fig. 6 e–g: Atrophic stage of ACA Fig. 6e: ACA – Purple colouring and atrophy on the back of the right hand and little finger, and purple blotches, stripes and infiltrates dorsally on the right knee Fig. 6f: ACA with ulnar stripes and purple blotches on the right underarm, and pronounced purple fibrous nodules below the elbow Fig. 6g: ACA with dark red to purple colouring and atrophy of the right hand dorsally (so-called “baked apple skin”) with swelling of the finger joints

Acrodermatitis initially manifests itself as pink reticular, then increasingly purple, oedematous infiltrated cushion-like erythemas, mostly on the extremities. The skin is inflamed, however there is initially no pain except for a feeling of heaviness of the extremity. This is the oedematous infiltrative stage of acrodermatitis chronica (Figure 6a and 6b (Fig. 6)). These purple infiltrates can also appear on the face and be confused with lupus erythematosus or a cutaneous malignant lymphoma [70].

##### Atrophic stage of ACA

In the course of the infection there is an increasing atrophy of all skin layers and skin appendages. Occasionally juxta-articular rough fibroid nodules and band-shape stripes appear (Figure 6f (Fig. 6)), e.g. rare but typical inflammatory ulnar stripes and swelling in the heel and Achilles tendon, or in other joints around the ACA (Figure 6b (Fig. 6)). This results in circumscribed fibrosis or pseudo-scleroderma in the area of the ACA which can be confused with circumscribed scleroderma. Arthritides, arthralgia and myalgia in the affected extremities are frequently associated with ACA [81]. 

A peripheral neuropathy occurs in 40–60% of patients in association with ACA. It is characterized by a feeling of numbness, a tingling sensation, burning and an increased sensitivity to pain (allodynia) [85], [86], [87]. 

Without antibiotic treatment living Borrelia can be detected for years in the skin and in the fibroid nodules [88]. In the course of the infection, all of the affected skin becomes atrophic and there is a loss of body hair, connective tissue and fatty tissue (Figure 6e and 6g (Fig. 6)). When the changes to the ACA-affected skin are symmetrical, they are difficult to differentiate clinically from age-related skin atrophy, acrocyanosis and chronic venous insufficiency. From a histological standpoint, acrodermatitis chronica atrophicans is characterised by a pronounced perivascular plasma-cell rich inflammatory infiltrate in all layers of the skin (Figure 6d (Fig. 6)) and, in the late stage, by an increasing atrophy of the epidermis, connective tissues and fatty tissues [89].

An ACA diagnosis is based on a typical clinical presentation, a typical histology and, as a rule, a high elevation of Borrelia IgG antibodies in the serum [81]. In unclear cases, particularly in the case of marginal elevation of antibody concentrations, the diagnosis has to be made by skin biopsy for histology and Borrelia DNA detection by NAT (PCR), or if possible through the cultivation of Borrelia from the skin. 

**Significant clinical features of acrodermatitis chronica atrophicans (ACA)**

- Initial oedematous infiltrative stage (plasma-cellular dermatitis) reddish colouring of the skin, mostly on one extremity
- Transition to the atrophic stage in the course of the disease, purple to brown colouring of the skin, skin atrophy, loss of body hair, connective and fatty tissues, emergence of veins, juxta-articular fibrous nodules and joint involvement
- Association with a peripheral neuropathy in around 50% of the cases
- Older women more strongly affected (Strong consensus: 19/19)

### 6.4 Manifestations in the nervous system and joints associated with cutaneous borreliosis 

An acute neuroborreliosis can simultaneously appear as part of early-stage borreliosis with erythema migrans. Arnez et al. found pleocytosis in the cerebrospinal fluid of 26% of the 214 children diagnosed with multilocular erythemata migrantia. Of these, 11% had clinically symptomatic lymphocytic meningitis [90]. Radiculoneuritis with characteristic nightly pain can also occur – in rare cases with paresis of the cranial nerves or peripheral nerves. 

A peripheral neuropathy of the affected extremity occurs in 50% of patients with ACA [87].

Rheumatic symptoms, above all myalgia and arthralgia, can occur in relatively early stages of the disease alongside erythema migrans. Cardiac symptoms with dysrhythmia (AV block) should be watched for, which can occur during or after erythema migrans. 

Lyme arthritis can either be the initial symptom or it can occur after a non-treated case of erythema migrans. Frequently the joint adjacent to the erythema migrans is affected. This manifests itself as acute intermittent arthritis with voluminous, at times, painful joint swelling, usually as mono or oligoarthritis. The knee joints are affected in 85% of the cases. The often massive swelling of the knee leads, unusually frequently and early on, to the development of popliteal cysts (Baker’s cysts). Ankle and elbow joints are less often affected, and almost never finger joints, especially in the form of a polyarthritis, have been observed. Lyme arthritis usually proceeds episodically, in other words, with repetitive inflammatory flare-ups that are interrupted by intervals of light to no symptoms. 

### 6.5 Differential diagnoses for cutaneous Lyme borreliosis 

The most frequent differential diagnoses for cutaneous Lyme borreliosis are listed in Table 2 (Tab. 2). 

**The variety of differential diagnoses shows that, except for typical erythema migrans, most of the cutaneous manifestations of Lyme borreliosis require careful dermatological diagnostic procedures.** In particular, the lack of response to antibiotic treatment should not be uncritically interpreted as persistent borreliosis and treated for months with antibiotics. 

It is, therefore, recommended to refer a patient with indistinct skin afflictions that persist after treatment to dermatologists or to dermatologically experienced paediatricians. 

**Recommendation:**

- Skin inflammations that were diagnosed as Lyme borreliosis and which have not healed after lege artis antibiotic treatment shall be referred to a dermatologist. (Strong consensus 11/12)

## 7 Diagnostics

### 7.1 Indirect pathogen detection (serodiagnostics, detection of antibodies) 

Due to the complex characteristics of the pathogen, indirect pathogen detection using serological methods continues to play a pivotal role in the diagnosis of Lyme borreliosis in practical laboratory-based medical care. In accordance with the methods and standards required in Germany, the antibodies are detected in a serum using a **two tiered diagnostic approach** with a standardised **screening test** (immunoassay: ELISA, CLIA etc.) and a **confirmation assay** (immunoblot). This is to ensure that the diagnostic procedure has a uniformly high level of sensitivity and specificity (Table 3 (Tab. 3)). 

In Europe, diagnostics tests for borrelial serology do not undergo any form of mandatory, extensive or independent clinical evaluation as part of the approval process. Thus a range of different test formats are on the market. In addition to various types of immunoassays, there are also a variety of test antigen preparations that use native and recombinant antigen combinations with, at times, different performance data. This partly explains the high degree of variability in the lab results which depend on the manufacturer and the test [12], [91]. Even though the principle testing procedures and the interpretation of serological test results are laid down as part of binding standards [92] in Germany, the interpretation of testing results, and in particular the evaluation criteria for immunoblot testing, are subject to manufacturer-dependent differences and have to be done in accordance with the respective manufacturer requirements as a result of the variability and insufficient standardisation of commercial test systems. This ongoing issue of insufficient testing standardisation is confirmed through meta-analytical investigations as part of external quality controls [12], [93]. In this respect, attending physicians should be aware of the qualifications of their diagnostic laboratory and the diagnostic assays and test specifications which it uses. 

#### The course of the immune response and interpretation of the findings

In the course of a natural infection, specific **IgM antibodies** are usually detectable 3–6 weeks after the onset of the illness; **IgG antibodies** reach their peak more slowly (weeks to months). 

It should be further noted that, after early, **successful** treatment of early manifestations, seroconversion can fail to appear under certain circumstances or, in the case of a positive detection of IgM antibodies, there doesn’t have to be a regular continuation of the immune response in the sense of a conversion from IgM to IgG. In contrast to textbook examples of the courses of immune response for many viral diseases, the antibody response to Lyme borreliosis often regresses very slowly both after an infection that is latent or cured, and after successful treatment. Thus, under certain circumstances, IgM reactivities or specific IgG values after such infections can remain detectable for months or even years. Often low positive borrelial-specific antibody values are a sign of a previous infection in the sense of **persisting antibodies from a past infection (serological scar)** [91]. However, a reinfection cannot be excluded in the case of such a lab result. Such findings have been detected in 20% of the people examined in serial investigations who belong to population groups that are frequently exposed, e.g. forestry workers, without there being or having been any symptoms of illness [94], [95]. Possible coincidences of these types of titres, with persisting antibodies from previous infections and unspecified findings, are also possible amongst the normal population [13], [96] and can be responsible for erroneous interpretations and diagnoses.

**Detection of elevated IgM antibodies only (without IgG) effectively excludes a late manifestation of Lyme borreliosis in the case of immune-competent patients.**

Diagnostic use of very sensitive early-phase antigens, such as VlsE, which enable the detection of a specific IgG response very early on in the course of the infection, means specific IgM antibody findings as part of Lyme borreliosis diagnostics are playing an increasingly limited role, especially since the IgM detection exhibits a poorer overall specificity than the IgG detection [97], [98]. However positive IgG findings can persist, in part, in high concentrations over longer periods of time so that no conclusions can be drawn regarding the activity of Lyme borreliosis or even the necessity for treatment in the absence of a classic activity marker, without additional clinical information and only on the basis of positive serological findings. At the same time, a statement can only be made about the significance of changes in findings if the comparison tests are carried out on serum samples that were taken at different times, ideally using a parallel approach as with the preserum, but, in any case, using the same test [91], [99].

An analysis using immunoblot within the framework of the stepwise diagnostic approach generally serves to not only specifically confirm the findings of the screening test, it also enables the immune response to be divided into an early and late stage so that a better correlation can be made between the lab findings and the clinical symptoms based on the characteristic band spectrum, particularly in the IgG immunoblot. Thus a **narrow spectrum of bands** with **antibodies against early-phase antigens (e.g. VlsE, OspC, p41)** is typically compatible with an early manifestation (e.g. erythema migrans, facial paresis) or a brief latent infection. However, it does not point to persistent clinical symptoms [91], [99], [100], [101]. In contrast, **a wide band spectrum, including reactions to late-phase antigens (e.g. p100, p17/p18)**, fits in well with a **late manifestation** (e.g. arthritis, acrodermatitis) [100], [101], also with an asymptomatic persistence of antibodies (serological scar), however it primarily does not point to an early manifestation or a short course of infection. **Reinfections** are difficult to detect based only on serological test results without additional clinical information and can only be detected based on a clearly verifiable **IgG increase** in a parallel approach, or **significant changes in the immunoblot band pattern** in serum samples that are tested in parallel. 

**One major premise of serological testing for Lyme borreliosis is the fact that the referring physician needs to be aware that these types of tests should only be requested when there is reasonable clinical suspicion.** Only when there is sufficiently high pre-test probability (prevalence of Lyme borreliosis in the patient cohort being investigated >20%) can a sufficiently utilisable positive predictive value of a positive test result even be assumed [102]. If the test is only ordered to exclude Lyme borreliosis in the case of unspecified or non-typical disease symptoms, the positive predictive value of the lab test drops to almost zero with respect to the possible confirmation of Lyme borreliosis. On the other hand, due to the relatively low overall prevalence and incidence of Lyme borreliosis in the general public, a negative test result, which excludes the disease in immune-competent patients with persisting symptoms, has an excellent negative predictive value. 

**Recommendation:**

- Serological diagnostics shall only be ordered when there is sufficient clinical suspicion. (Strong consensus: 19/19)
- The diagnostics shall be conducted using a stepwise approach (screening test and confirmation test). (Consensus: 16/19)
- Positive antibody detection is not proof of a clinically present Lyme borreliosis. (Strong consensus: 19/19)
- Negative antibody detection almost entirely excludes Lyme borreliosis in healthy immune system patients with a protracted duration of illness. (Consensus: 16/19)
- An isolated positive IgM detection argues against a late manifestation of Lyme borreliosis. (Consensus: 17/19)

**Dissenting opinion (German Borreliosis Society)**

- There are no systematic studies on the frequency of Bb antibodies in the case of late-stage Lyme borreliosis. The view that an isolated IgM detection argues against a late manifestation of Lyme borreliosis has not been verified by the literature.

### 7.2 Direct pathogen detection 

The respective microbiological diagnostic quality standards (MiQ Lyme borreliosis, MiQ PCR) apply in the direct pathogen detection of Lyme borreliosis using culture and PCR. 

#### 7.2.1 Culture

Direct detection by culture with the modified Barbour-Stoenner-Kelly medium is considered to be the gold standard and to be clear proof of an infection with *B. burgdorferi* [103], [104]. Direct detection of skin manifestations by culture are frequently successful. To a limited degree, detection by culture is also possible in liquor and, in very rare cases, in synovial fluid, synovial biopsies and blood. In individual cases, the detection of *B. burgdorferi* has also been achieved in other tissue samples, e.g. heart muscle and iris [105], [106]. Cultivating from patient samples using suitable media is time-consuming and materially intensive, and usually takes more than two weeks. The sensitivity of the methods in European studies is between 40% and 90% for erythema migrans and between 20% and 60% for ACA [107], [108], [109], [110]. Overview in: [111]. Because of the invasiveness of the sample taking, direct detection by culture should therefore be based on a clear indication and explicitly remain limited to specially identified reference laboratories, such as the National Reference Centre for Borrelia at the Bavarian State Office for Health and Food Safety in Oberschleissheim. In addition, further molecular-biological confirmation assays are required in positive cases. 

**Recommendations for direct detection by culture:**

- Direct detection by culture should only be used in differential-diagnostically ambiguous cases. (Strong consensus: 19/19)
- The cultivation of *Borrelia burgdorferi* sensu lato should be limited to specialist laboratories. (Strong consensus: 19/19)
- Positive culture results are to be confirmed using suitable molecular-biological methods. (Strong consensus: 18/19)

#### 7.2.2 Direct detection using molecular-biological detection methods 

The detection methods currently being used in Lyme borreliosis diagnosis should be regarded as having a low level of standardisation [112]. This applies to DNA isolation from suitable clinical materials, as well as to the reaction conditions and the selection of the reaction starter molecules (primers). In principle, the detection of Borrelia from a skin biopsy using nucleic acid amplification techniques (NAT, usually PCR) is very reliable and, in the case of early manifestations, is more sensitive than serological antibody detection. The diagnostic sensitivity of NAT is around 70% for detection from biopsies from erythema migrans and acrodermatitis chronica atrophicans [107], [113], [114], [115], [116]. However, positive results have to be confirmed through molecular-biological confirmation assays with regard to specificity (probe hybridisation, sequencing of the amplificate) and the results must be indicated in the findings. 

After treatment, Borrelia DNA can still be detected for weeks – or even months – in the affected area of skin before conclusions can be drawn as to whether the therapy has failed [117], [118], [119]. Molecular-biological detection of pathogens without the simultaneous presence of typical disease manifestions is not clinically relevant. Direct molecular-biological detection from urine samples is not currently recommended due to ambiguous diagnostic sensitivity and specificity [113], [120], [121]. Because of the invasiveness of the sample taking, direct detection by culture should therefore be based on a clear indication (e.g. unexplained skin manifestation that has been differentially diagnosed) and explicitly remain limited to specially identified reference laboratories, such as the National Reference Centre for Borrelia at the Bavarian State Office for Health and Food Safety in Oberschleissheim. In addition, further molecular-biological confirmation assays are required in positive cases. 

**Recommendations for direct molecular-biological detection: **

- Direct molecular-biological detection (PCR) is not a screening test if there is suspicion of Lyme borreliosis. (Strong consensus: 19/19)
- A negative PCR test result does not exclude Lyme borreliosis. (Strong consensus: 19/19)
- A positive PCR test result shall be confirmed by further molecular-biological methods and the detected genospecies shall be indicated in the findings. (Strong consensus: 19/19)
- A positive PCR test result after treatment with antibiotics in accordance with the guidelines or without typical clinical manifestation has no clinical relevance. (Consensus: 16/19)
- Direct molecular-biological detection should be limited to ambiguous skin manifestations and reserved for specially identified microbiological laboratories. (Strong consensus: 20/20)

### 7.3 Diagnosis of clinical skin manifestations 

#### 7.3.1 Erythema migrans (typical)

**Figure 7:**
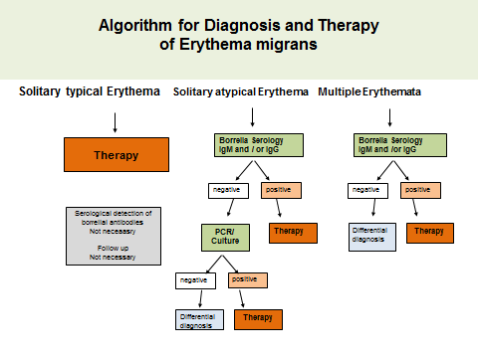
Algorithm for diagnosing a solitary or multilocular erythema migrans

If a clinically typical erythema migrans is present (see section on clinical manifestations) no further laboratory diagnostic confirmation needs to occur; antibiotic treatment should begin immediately (Figure 7 (Fig. 7)). 

**Recommendation:**

- If a typical erythema migrans is present (see section on clinical Manifestations) no further laboratory diagnostic confirmation (serological, cultural, molecular-biological) needs to occur. (Strong consensus: 20/20)
- If a typical erythema migrans is present, antibiotic treatment shall begin immediately. (Strong consensus: 20/20)

#### 7.3.2 Erythema migrans (atypical)

If an **atypical erythema migrans** is suspected, antibody and pathogen detection by PCR and culture is available. A serological test should be carried out in every case. If the findings remain ambiguous, the aim should be pathogen detection using PCR, if necessary also by culture (Figure 7 (Fig. 7)). A skin biopsy should be taken near the inflamed edge. After informing the patient and obtaining written consent, the selected area of the skin is numbed using local anaesthesia. After thorough disinfection of the skin, a 4 mm punch is used to remove the skin, which is put in a sterile vessel with 0.9% saline solution. Direct inoculation in the cultivation medium only makes sense when the sample can be processed in the lab within a few hours. Otherwise, fast growing skin bacteria can hamper the cultivation of the Borrelia. 

A histological analysis rarely has a guiding nature in the case of erythema migrans. It can, however, make sense for differential-diagnostic clarification. 

**Recommendation:**

- In the case of an atypical clinical appearance of erythema migrans, suspicion shall be clarified through a serological test. (Consensus: 18/20)
- If the serological test is negative and the clinical suspicion remains, direct cultural or molecular-biological detection from biopsy material shall be used for clarification. (Strong consensus: 20/20)

#### 7.3.3 Multiple erythemata migrantia (MEM)

If multiple erythemata migrantia, also known as multilocular erythema migrans (**MEM**) is suspected, serological antibody detection and pathogen detection using PCR and culture from a skin biopsy are available. A serological test should be carried out in every case. If the findings remain ambiguous, the aim should be to detect the pathogen using PCR, if necessary also by culture (Figure 7 (Fig. 7)) (see 7.2.1). Clinical signs of extra-cutaneous symptoms should be watched for in the case of MEM (see Table 1 (Tab. 1) in the section on clinical manifestations). 

**Recommendations:**

- A serological test shall be carried out when MEM is suspected. (Strong consensus : 20/20)
- If the serological test is negative and the clinical suspicion remains, direct cultural or molecular-biological detection in biopsy material shall be used for clarification. (Strong consensus: 20/20)

#### 7.3.4 Borrelial lymphocytoma

Confirming the diagnosis through serological antibody detection is obligatory and, in most cases, antibodies against *B. burgdorferi* can be detected [38], [70], [122]. When the findings are still ambiguous, the patient shall be referred to a dermatologist in order to detect the pathogen by PCR or, if necessary, culture. Two skin biopsies should be taken from the abnormal skin (see 7.2.2): one for the histological test in a 4% formalin solution, and one for the culture and PCR test in sterile, physiological saline solution. 

PCR and culture allow *B. burgdorferi* to be detected. Although there is limited data on the sensitivity of the methods used to identify borrelial lymphocytoma; a PCR detection success rate can be expected in around 70% of the cases [73], [122]. 

**Recommendation:**

- If there is an unambiguous clinical presentation of borrelial lymphocytoma and a positive serology, further microbiological tests are not required. (Strong consensus: 20/20)
- If there is an unambiguous clinical presentation of borrelial lymphocytoma, antibiotic treatment shall begin immediately. (Consensus 16/20)
- If the clinical presentation is not unambiguous and the serology is negative, further tests (primarily histology, molecular-biology, possibly culture) shall be conducted for differential-diagnostic clarification. (Strong consensus 20/20)

#### 7.3.5 Acrodermatitis chronica atrophicans

If ACA is clinically suspected, a Borrelia serology should be carried out first. High antibody values in the IgG screening test, combined with a broad spectrum of borrelial-specific bands in the IgG blot or similar tests (see Section 7.1), are indications of ACA. A negative IgG serology excludes, with high certainty, ACA in immune-competent patients. 

In ambiguous cases the patient should be referred to a dermatologist for differential diagnosis. If uncertainty remains, two skin biopsies (see 7.2.2.) should be taken from the abnormal patch of skin: one for the histological test in a 4% formalin solution, and one for the culture and PCR test in physiological saline solution. In the case of ACA, *B. burgdorferi* DNA can be detected in around 70% of the cases. 

**Recommendation:**

- When ACA is clinically suspected, the diagnosis shall be confirmed through a serological test. (Strong consensus: 19/20)
- High IgG antibody values in the screening test, combined with a broad band pattern in the IgG immunoblot test, indicate a suspected clinical diagnosis. (Strong consensus: 20/20)
- A negative Borrelia serology excludes ACA with a high degree of certainty in immune-competent patients. (Strong consensus: 20/20)
- The diagnosis shall be histologically confirmed in all cases. (Majority approval: 12/20)
- When the clinical picture is ambiguous, further diagnostic clarification through biopsy and subsequent histological testing should be done. When the findings are unclear, direct detection by culture and molecular biology is recommended. (Strong consensus: 19/20)

#### 7.3.6 Ambiguous dermatological pathologies with a suspicion of Lyme borreliosis 

See Table 4 (Tab. 4). 

**Recommendation:**

- If a cutaneous manifestation of Lyme borreliosis is suspected and there is no unambiguous clinical presentation, a skin biopsy with a histological examination shall be conducted along with direct pathogen detection using culture and molecular-biological methods. (Strong consensus: 20/20)

### 7.4 Non-recommended diagnostic approaches 

In addition to the traditional diagnostic methods listed above, which are used when Lyme borreliosis is suspected, the literature describes a whole series of diagnostic techniques that, in part, have been inconclusively evaluated. This includes the immuno-histochemical detection of *B. burgdorferi* in biopsies and of antigens from blood and urine, as well as functional tests that test for cellular immunity (lymphocyte transformation tests (LTT), cytokine detection). Currently there is a lack of scientific investigations that prove there is a diagnostic benefit. Because the available LTT methods lack specificity, they should not be used. 

**Methods that are not recommended for use in the diagnosis of cutaneous manifestations of Lyme borreliosis:**

- Immunohistochemical detection of Borrelia from tissue is currently not recommended. (Strong consensus: 19/19)
- The lymphocyte transformation test (LTT) and the detection of specific cytokines is currently not recommended. (Strong consensus: 18/19)
- Detection of Borrelia in engorged ticks is not recommended. (Strong consensus: 19/19)
- Detection of Borrelia antigens from patient samples is currently not recommended. (Strong consensus: 19/19)
- Direct detection of Borrelia in patient samples using light microscopy is currently not recommended. (Strong consensus: 18/19)
- The detection of circulating immune complexes is currently not recommended. (Strong consensus: 18/19)

### 7.5 Quality control and quality assurance 

According to the guidelines of the German Medical Association (Bundesärztekammer), diagnostic laboratories must currently participate in infection-related serological round robin tests twice a year. This applies to serological antibody detection and to direct molecular-biological detection of Borrelia when Lyme borreliosis is suspected. The results of the external quality assessment tests (EQA tests), which INSTAND e.V. has been carrying out for years, reveal extensive heterogeneity in the testing systems currently on the market. The pass rates for the conventional serological and molecular-biological test systems, which have been collected from meta-analytical data, show that, despite good analytical pass rates for immunoassays and molecular-biological tests, clinical diagnostic interpretation of the result constellations often proves difficult and can hamper medical treatment in daily clinical practice [12], [91]. Thus, when Lyme borreliosis is suspected, infection diagnostics are to be conducted in laboratories that meet the laboratory diagnostic standards in accordance with the diagnostic guidelines of the expert medical societies and the guidelines of the German Medical Association. These laboratories must regularly and successfully participate in external quality assurance tests (round robin tests). Physicians treating patients with Lyme borreliosis should query about and ensure that these prerequisites are met in the laboratories charged with carrying out their diagnostic testing. If questionable result constellations or implausible test results are produced, expert laboratories specialising in Lyme borreliosis diagnostics and the National Reference Centre for Borreliosis at the Bavarian State Office for Health and Food Safety in Oberschleissheim should be consulted. 

**Recommendation:**

- Attending physicians shall be aware of whether their diagnostic laboratory complies with the respective diagnostic standards and qualifications and the extent to which the diagnostic assays used there conform to guidelines. (Strong consensus: 18/19)

## 8 Treatment of cutaneous Lyme borreliosis

Recommendations for treating Lyme borreliosis have been published in numerous European and American guidelines since 2004 (see Annex 2 “Comparison of Guidelines and Therapies” in Attachment 1).

Table 5 (Tab. 5) summarises the best-evaluated antibiotic therapies taken from American and European guidelines. 

**Doxycycline and amoxicillin** are the antibiotics of choice in all guidelines. 

Both antibiotics are very effective in the dosages listed in Table 5 (Tab. 5) and are usually tolerated well. Gastrointestinal complaints can occur during treatment with doxycycline. It is particularly important that they are not taken together with dairy products. Furthermore, patients should be informed of the risk of phototoxic skin reactions and use light stabilisers when taking the antibiotics. 

During treatment with amoxicillin, non-allergenic skin exanthemas frequently appear on the 8^th^ day on the torso. If they are light exanthemas, the treatment can continue. If itching occurs, symptoms can be treated with antihistamines and skin care products. Corticosteroids are not necessary. 

Of the oral cephalosporins, only **cefuroxime axetile** has demonstrated an efficacy that is comparable to treatment with doxycycline and amoxicillin [123]. The absolute bioavailability of cefuroxime axetil is comparatively low (40–45%). The best resorption is achieved when it is taken directly after a meal. 

Other 1^st^ and 2^nd^ generation cephalosporins are not effective enough [124]. 

In the case of disseminated early-stage infection, intravenous treatment with ceftriaxone does not achieve any better results than oral doxycycline treatment [125].

Of the macrolides, **azithromycin** has proven to be adequately effective [79], [126], [127], [128]. The long tissue half-life period is advantageous because of the long generation time of Borrelia. The efficacy of **clarithromycin** is regarded as controversial. Clarithromycin was compared with amoxicillin in one of the newer, open, randomised comparative studies of children with erythema migrans and was classified as equally effective [129]. Roxithromycin is not effective enough. Because of its uncertain resorption and indications of resistance, erythromycin is no longer a treatment of choice [28], [130]. 

Treatment with oral penicillin V is controversial. Austrian, Swedish and Slovenian studies show that it is sufficiently effective [90], [131], [132], [133]. 

**It is particularly important that dosage and length of treatment are observed. **

**Cutaneous early manifestations** should be treated for 10–21 days (Table 5 (Tab. 5)). The length of treatment depends on the duration and severity of the clinical symptoms; in the case of solitary erythema migrans without general symptoms, a 10 to 14 day treatment is sufficient. In a comparative study by Stupica et al. [134] the results of treating localised erythema migrans with doxycycline for 10 versus 14 days were evaluated. There were no differences in the way the erythema healed. In both treatment groups symptoms persisted no longer or more frequently than in healthy subjects. Treatment should last 21 days if there is evidence that the Borrelia has disseminated (indicated by a flu-like feeling), or in the case of multiple erythemata migrantia and borrelial lymphocytoma. 

Taking doxycycline or amoxicillin orally for 30 days to treat **cutaneous late manifestations** (acrodermatitis chronica in the oedematous-infiltrative or atrophic stage) without neurological involvement is usually sufficient [81], [135]. However, if there are also neurological symptoms, intravenous treatment with penicillin G or 3^rd^ generation cephalosporins ceftriaxone or cefotaxime may be necessary. 

The cure rates – defined as the reinstatement of the body’s original condition with regression of the disease-specific symptoms after successful treatment – is between 95%–100% when the localised and disseminated early manifestations are treated in time [136], [137]. 

Treatment failure with evidence of the pathogens after therapy rarely occurs if the treatment is conducted lege artis [45], [138]; individual cases have been published [104], [105], [139], [140], [141], [142]. 

Two larger studies were able to show that new infections with other Borrelia strains were the reason why Lyme borreliosis returned in every case [143], [144]. 

Currently there are no indications of a development of secondary antibiotic resistance of *B. burgdorferi* to the antibiotics recommended in the guidelines [145], [146], [147], [148].

If the late manifestations remain untreated for a long period of time, there is a higher risk of the patient having persistent physical symptoms and of their skin, joints and nervous system not properly healing. 

It is disputed whether repeated antibiotic treatment makes sense for these patients with persisting complaints. According to published randomised controlled trials (RCT), long-term antibiotic treatment is less than promising [149], [150], [151], [152], [153], [154]. 

A European RCT published in 2016 (PLEASE Study) looked at 280 patients whose complaints persisted for more than 2 years after their Lyme borreliosis had been treated with antibiotics (78 patients after erythema migrans, 15 patients after meningoradiculitis) and 153 seropositive patients with borreliosis-related complaints after a tick bite. The study compared the health-related effects of a 2-week compared to a 14-week round of antibiotics. First, all of the patients that had previously been treated with antibiotics were given 2 g of ceftriaxone i.v. for 2 weeks. Then the patients were randomly placed in 3 groups. Group 1 received doxycycline 200 mg/d p.o. for 12 weeks, Group 2 clarithromycin 2x 500 mg plus hydroxychloroquin 2x 200 mg/d for 12 weeks, and Group 3 a placebo for 12 weeks. Treatment success was assessed as health-based quality of life after 14 weeks and then up to 52 weeks using the RAND 36 Health Status Inventory. The aggregate score improved equally after treatment in all three groups without a significant difference. The assessment of the quality of life remained lower than in the general population in all three groups. No difference in treatment success between the short-term treatment and the two long-term treatments could be made. Patients receiving the long-term treatment had considerably more antibiotic-related side-effects (primarily photosensitivity (18.6%) and nausea (10.5%) in connection with doxycycline, and primarily nausea (10.4%), diarrhoea (9.4%) and allergic exanthemas (8.3%) in connection with clarithromycin/hydroxychloroquine.) Vision problems were the most frequent complaint of the placebo group (10% of the patients) [155], [156].

### 8.1 Treatment during pregnancy and nursing 

Oral treatment with amoxicillin p.o. is recommended during pregnancy and nursing. Alternatively, penicillin G and ceftriaxone can be administered i.v. [157], [158]. If the patient has an identified allergy to penicillin, azithromycin or cefuroxime axetil can be prescribed after strong indication. Ceftriaxone can be taken intravenously under clinical surveillance since the risk of a cross allergy between penicillin and 3^rd^ generation cephalosporins is around 1% [159].

### 8.2 Treatment of children

Children can be treated with 4 mg/kg KG/day (up to a maximum dosage of 200 mg/day) of **doxycycline** once their tooth enamel has completely formed at age 9 and over (>8 years). For children under 8, the treatment of choice is 50 mg/kg KG/day of **amoxicillin** (Table 5 (Tab. 5)). Taking it the required 3 times a day can be difficult for kindergarten- and school-aged children. 

Alternatively, **cefuroxime axetil** 30 mg/kg KG/day, **azithromycin** 5–10 mg/kg KG/day or **clarithromycin** 15 mg/kg KG/day can be prescribed, which is taken twice daily [129].

### 8.3 Therapy adherence

In order to improve **treatment adherence/therapy compliance**, the patient should be informed before beginning the treatment of the aspects of taking prescribed antibiotics and the potential risks of undesired effects. 

A frequent cause of treatment failure is the incorrect administration of doxycycline. It should be noted that resorption can be compromised when it is taken together with bivalent or trivalent cations, such as aluminium, calcium (milk, dairy products and fruit juice containing calcium), and magnesium, in antacids or through iron supplements, as well as through activated charcoal and colestyramine. Therefore, there should be a 2 to 3 hour time span between when the antibiotic is taken and the medicine or food is ingested. 

Another reason for treatment failure is irregular administration e.g. forgetting to take the midday dose in the case of amoxicillin, or when the length of antibiotic treatment is insufficient e.g. due to a deterioration in symptoms as a result of a Herxheimer reaction, because of gastrointestinal complaints, or as a result of phototoxic skin reactions through increased sensitivity to light from doxycycline. 

In the case of a disseminated infection, the patient should be informed about a possible **Herxheimer reaction** with a flare up of the erythemas, which occurs in approx. 10% of cases, a feeling of being very unwell, and a rise in temperature in approx. 2% of cases within 24 hours of taking the antibiotics [78], [129]. Occasionally this reaction is delayed. It is a temporary immunological reaction as a result of the upregulation of proinflammatory cytokines and can be treated, for example, with non-steroidal anti-inflammatory drugs (NSAID). Cortisone treatment is not necessary. The antibiotic should continue to be taken. 

**Recommendations for treating cutaneous Lyme borreliosis: **

**Antibiotics**

- Doxycycline or amoxicillin p.o. are the treatments of choice (Strong consensus: 17/18)
- Treatment alternatives are cefuroxime, azithromycin, possibly also clarithromycin p.o. (Strong consensus: 17/18)
- Ceftriaxone i.v. is the treatment of choice for cutaneous Lyme borreliosis with neurological manifestations (Majority approval: 10/19)
- See the recommendations of other expert medical societies for antibiotic treatment of patients with cutaneous Lyme borreliosis with neurological or cardiological manifestations. Possibilities include ceftriaxone i.v., cefotaxime i.v., penicillin G i.v. or doxycycline p.o. (Strong consensus: 18/18)

**Duration of treatment**

- The treatment of the early manifestations of cutaneous Lyme borreliosis shall last 14–21 days. (Exceptions are azithromycin 5–10 days; doxycycline 10–14 days in the case of solitary erythema migrans) (Consensus: 17/19)
- The treatment of cutaneous late manifestations shall last 30 days. (Consensus: 17/19)
- A general extension of treatment beyond the recommended amount of time is not recommended. (Consensus: 17/19)
- Treatment can be extended in individual cases depending on the clinical progression and after a re-evaluation of the diagnosis. (Consensus: 17/19)
- Treatment is renewed on a case-by-case basis when the pathogen has been confirmed. (Consensus: 16/19)
- The diagnosis should be re-evaluated if the cutaneous symptoms persist or progress despite treatment with antibiotics in line with the guidelines. (Consensus: 17/19)

**Recommendations for treating cutaneous Lyme borreliosis during pregnancy:**

- Amoxicillin p.o. shall be administered as the treatment of choice during pregnancy. (Strong consensus: 18/18)
- Penicillin G i.v. and ceftriaxone i.v. represent alternative therapies during pregnancy. (Strong consensus: 18/18)
- If the patient is allergic to penicillin, cefuroxime p.o., ceftriaxone i.v., cefotaxime i.v. or azithromycin p.o. should be used. (Consensus: 15/17)

**Recommendations for treating cutaneous Lyme borreliosis in children: **

- Amoxicillin p.o. shall be administered as the treatment of choice in children under 8. (Strong consensus: 17/17)
- Children aged 9 and over can take doxycycline p.o. (Strong consensus: 17/17)
- Azithromycin, clarithromycin or cefuroxime p.o. represent alternative treatments for children (Strong consensus: 17/17)

**Dissenting opinion (OnLyme Aktion) **

- When other causal factors can be excluded and cutaneous, illness-specific symptoms recur or do not regress, another suitable antibiotic can be considered, taking into account the patient’s individual situation.

**Dissenting opinion (German Borreliosis Society)**

- There are no evidence-based studies on the efficacy of treating late-stage Lyme borreliosis, particularly ACA, with antibiotics. The paper by Aberer et al. (1996) [135], cited in the text, states that the efficacy of ceftriaxone needs to be reviewed in further studies. In terms of oral antibiotics, it has been established that the length of treatment is a more critical factor than the type of antibiotic (penicillin/doxycycline).

### 8.4 Persisting symptoms after treatment/post-treatment Lyme disease syndrome (PTLS) 

After antibiotic treatment has been carried out in accordance with the guidelines, inflammatory reactions can persist and symptoms such as tiredness, joint and muscle pain, headaches, a general feeling of being unwell, irritability or paraesthesia can last for months. If the unspecific constitutional symptoms last for more than 6 months, it is considered by some authors to be post-Lyme syndrome (PLS) or post-treatment Lyme disease syndrome (PTLDS) [150], [160]. So-called PTLDS is a syndrome that has yet to be generally defined scientifically and therefore is not yet universally accepted. It can be diagnostically differentiated from diagnosed late manifestations of Lyme disease and symptoms resulting from persisting reproducible pathogens and from improper healing. The benefit of repeated and long-term treatment with antibiotics has not been verified. 

In a controlled study of patients with erythema migrans, in which a control group containing individuals of similar age and gender was simultaneously studied, no increased incidence of post-therapeutic symptoms compared to the control group were identified [136].

Several studies indicate special immunological characteristics. Patients who have persisting symptoms for months to years after receiving antibiotic treatment were identified as frequently having anti-neural antibodies [161], as well as a weaker Th1-immune response with elevated interleukin 23 concentrations in serum [39].

Bockenstedt et al. were able to identify Borrelia DNA in mice when treatment was focussed near cartilage, however they did not find any living Borrelia [117]. Persisting DNA and RNA, as well as living Borrelia, were detected in rhesus monkeys through xenodiagnoses (transfer of tissue to laboratory animals) [162]. Since these were animal studies, no statement on what this means for human infections can currently be made. 

### 8.5 Course of action for persisting skin changes and symptoms after antibiotic treatment 

A primary incorrect diagnosis is a common reason for persisting skin changes and symptoms after treatment with antibiotics [163].

In the case of clinically diagnosed erythema migrans and multiple erythemata migrantia that do not heal within 6 weeks, a differential diagnosis of circumscribed scleroderma (morphea), granuloma annulare, sarcoidosis, erythema annulare et diutinum, tinea (with low epidermal involvement) or uticarial vasculitis should be considered.

Patients should be referred to a dermatologist for further diagnostics. Borrelial lymphocytoma often heals very slowly over many months. According to studies by Maraspin et al. on 85 patients, healing time was, on average, 28 days (7–270 days). The longer the borrelial lymphocytoma was present, the longer it took to heal [73]. If the knots persist for more than one year, or new knots appear, a skin biopsy should be carried out by a dermatologist for a histological diagnosis and Borrelia PCR. Cutaneous pseudolymphoma, Jessner’s lymphocytic infiltration or a malignant lymphoma should be considered in the differential diagnosis. 

It takes years following antibiotic treatment for skin changes to slowly regress in the case of an acrodermatitis chronica atrophicans that has persisted for years. The atrophy of the skin, tissue and fat can be irreversible – especially in older people. This also applies to ACA-associated peripheral neuropathy. (See also the AWMF-S3 Guideline on Neuroborreliosis which is in progress.) 

Age-related skin atrophy, chronic thermal damage to the skin, e.g. chilblains and heat melanosis, as well as chronic venous insufficiency with stasis dermatitis can be considered in the differential diagnosis.

Chronic neuropathic pain after adequate antibiotic treatment of acrodermatitis chronica atrophicans with peripheral neuropathy is treated in accordance with the DGN’s guideline “Neuropathic Pain” (AWMF – guidelines register no. 030/114). 

All patients whose symptoms persist after antibiotic treatment of cutaneous Lyme borreliosis should undergo careful differential diagnostic clarification by respective specialists, above all, for internal medicine (infectiology, rheumatology, cardiology, endocrinology), psychosomatics, psychotherapy, psychiatry or palliative care, since chronic infections with another etiology, other internal medical disorders, autoimmune diseases, chronic pain syndrome, and depressive and somatoform disorders should also be considered in the differential diagnosis and need to be treated accordingly. 

**Recommendations for persisting symptoms after treatment in accordance with the guidelines: **

- **If an erythema or multiple erythemas persist after treatment of erythema migrans** (longer than 6 weeks), the patient shall be referred to a dermatologist for a differential diagnosis of circumscribed scleroderma (morphea), granuloma annulare, sarcoidosis, erythema annulare et diutinum, tinea or urticarial vasculitis. (Strong consensus: 17/17)
- **If a lymphocytoma persists or progresses after treatment** the patient shall be referred to a dermatologist for a differential diagnosis (cutaneous pseudolymphoma, Jessner’s lymphocytic infiltration or malignant lymphoma). (Strong consensus: 17/17)
- **If the acrodermatitis chronica atrophicans persists after treatment** the patient shall be referred to a dermatologist for further consultation and for a differential diagnosis (age-related skin atrophy, chronic thermal damage to the skin e.g. chilblains and heat melanosis, chronic venous insufficiency with stasis dermatitis). (Strong consensus: 16/16)

## 9 Prophylaxis

### 9.1 Preventing tick bites

The best prophylaxis is to prevent tick bites by wearing clothing that covers the body, and carefully checking the skin, including the scalp, after being outdoors. This is particularly important for children, who have an increased risk when playing outdoors between spring and autumn.

Insect repellents that are effective against ticks, e.g. diethyltoluamide (DEET), icaridin (1-(1-methylpropyl carbonyl)-2-(2-hydroxyethyl)piperidine), ethyl butylacetylaminopropionate (EBAAP, IR 3535) can also be used, however their effectiveness is limited to up to 4 hours [164], [165]. 

### 9.2 Preventing Lyme borreliosis

**Removing the ticks** before they become engorged with blood is very important. The risk of a Borrelia transfer increases with the length of time that the tick sucks [17]. Transmission within the first 12 hours has rarely been observed in laboratory animals. After being in a garden, park, field forest or meadow where there may have been contact with a tick, the body should be checked the same evening for ticks. 

The ticks should be removed immediately with a tick tweezer or a tick card in order to prevent the transfer of the Borrelia. If parts of the suction organ remain in the skin, they can later be removed with a needle or a curettage [160]. If the head or the suction organ remains in the skin, this is not critical in terms of a Borrelia transfer. When nymphs and adult ticks are engorged with blood, their bodies should not be squeezed in order to prevent a possible transfer of the Borrelia. 

Checking the tick that has been removed from the skin for borrelia does not make sense since the detection of the borrelia in the tick is not sufficiently predictive for whether the Borrelia has been transferred to the host and for the emergence of the disease. 

After removing the tick, the patient should be informed of the **necessity of observing the bite site** over the subsequent 6 weeks (Annex 1: “Patient information after a tick bite” in Attachment 1). 

### 9.3 Prophylactic treatment after a tick bite 

According to an American study, the risk of infection after a tick bite can be reduced through a one-time prophylactic administration of 200 mg of doxycycline (87% effectiveness) [166], [167]. The results, however, should be interpreted with caution since only one follow-up check took place after 6 weeks. Thus no statement can currently be made as to whether this is sufficiently effective with regard to a late infection.

In light of the low risk of infection, doxycycline would have to be administered unnecessarily many times in order to prevent a potential infection. According to projections of infection risk in endemic areas, 40–125 prophylaxes would have to be taken in order to prevent 1 infection [168]. Impact on the intestinal flora and a possible development of resistance through frequent prophylaxis is conceivable. Therefore, oral doxycycline prophylaxis in Europe is not recommended. 

The prophylactic application of an antibiotic cream is also controversial. Animal studies with azithromycin cream reveal a good prophylactic efficacy [169], [170]. Placebo-controlled studies on the effectiveness in humans have yet to be published. This treatment is not currently recommended due to the lack of clinical data. 

**Recommendations on infection prophylaxis:**

- **Clothing that covers the body should be worn to prevent tick bites.**
- Using tick repellents can be recommended with some reservations.
- Skin should be inspected in the evening for ticks after being outside in an area where there is a possibility of the individual coming into contact with ticks.
- Ticks should be removed early in order to prevent Lyme disease.
- The site of the bite should be observed for up to six weeks.
  (Consensus: 15/16)

**Not recommended:**

- Analysing the removed tick for Borrelia is not recommended. (Consensus: 15/16)
- Local or systemic prophylactic antibiotic treatment after a tick bite is not recommended. (Consensus: 14/16)

### 9.4 Vaccines 

No approved vaccine that can be used on humans is currently available.

A vaccination with recombinant lipidated Osp A has been evaluated in the USA as part of a major study and has shown to be effective [171], [172]. The vaccine has been approved in the USA since 1999; however, it was taken from the market by its manufacturer. The reason for this is not medical. Reports on undesired vaccine reactions in individuals who are genetically predispositioned were refuted by multiple qualified studies [173], [174], [175]. This monovalent vaccine is not suitable for Europe as it only protects against an infection with *B. burgdorferi* sensu stricto, and not against the genospecies *B. afzelii* and *B. garinii* that are frequently found in Europe.

A polyvalent Osp A vaccine is currently being developed for Europe [176], however approval is not expected in the foreseeable future. 

## Notes

### Procedure for forming a consensus 

The guideline was created using a modified Delphi process and was voted on in an extended consensus conference of the Interdisciplinary S3 Guideline Group, moderated by Prof. Ina Kopp, Head of the AWMF Institute for Medical Knowledge Management. 

It was passed by the 22 expert medical societies and patient organisations involved.

The guideline is a part of the registered Interdisciplinary S3 Overall Guideline on the “Diagnosis and Treatment of Lyme Borreliosis”.

### Support

This guideline was created without the influence or financial support of sponsors.

The funds required to create and to translate this guideline were provided by the German Society for Dermatology and the Society for Promotion of Quality Assurance in Medical Laboratories (INSTAND e. V.). 

Travel expenses were provided by the respective expert medical societies. 

### Declaration of competing interests by the authors 

Table in the Guideline Report, Section 5 (in German): http://www.awmf.org/uploads/tx_szleitlinien/013-044m_S2k_Kutane_Lyme_Borreliose_2016-05_01.pdf.

## Supplementary Material

Annex 1–4

## Figures and Tables

**Table 1:**
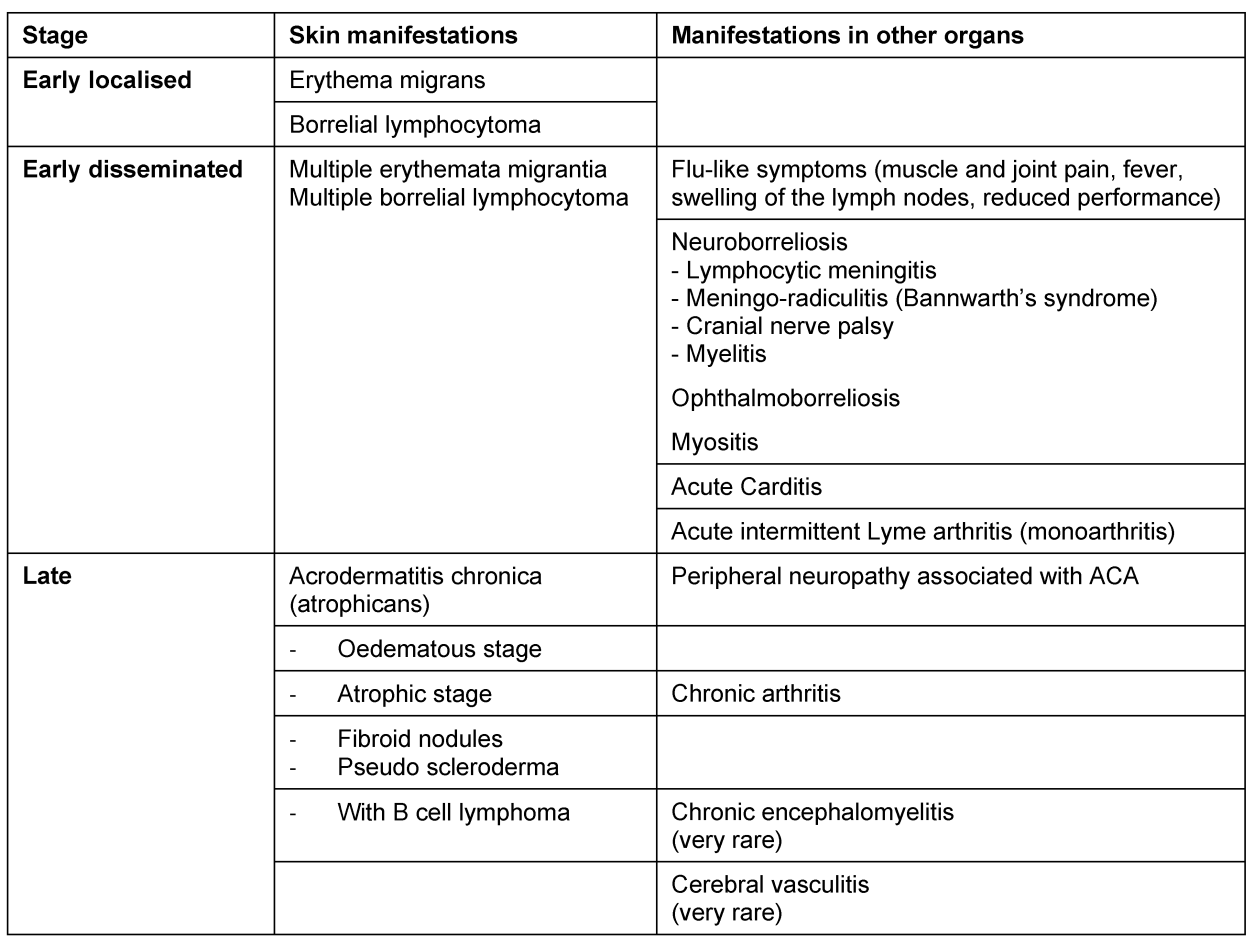
Clinical manifestations of Lyme borreliosis

**Table 2:**
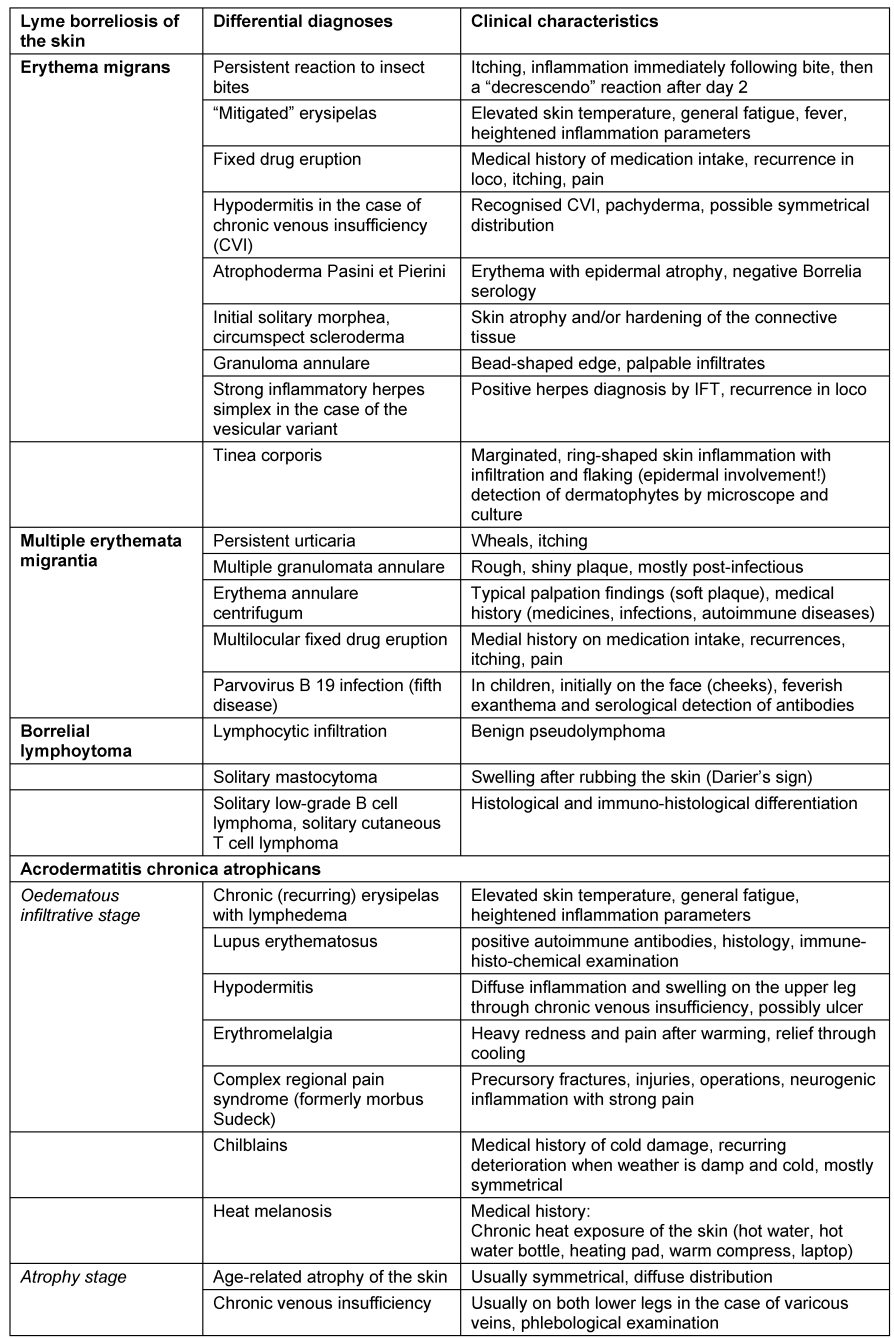
Clinical differential diagnoses of cutaneous Lyme borreliosis

**Table 3:**
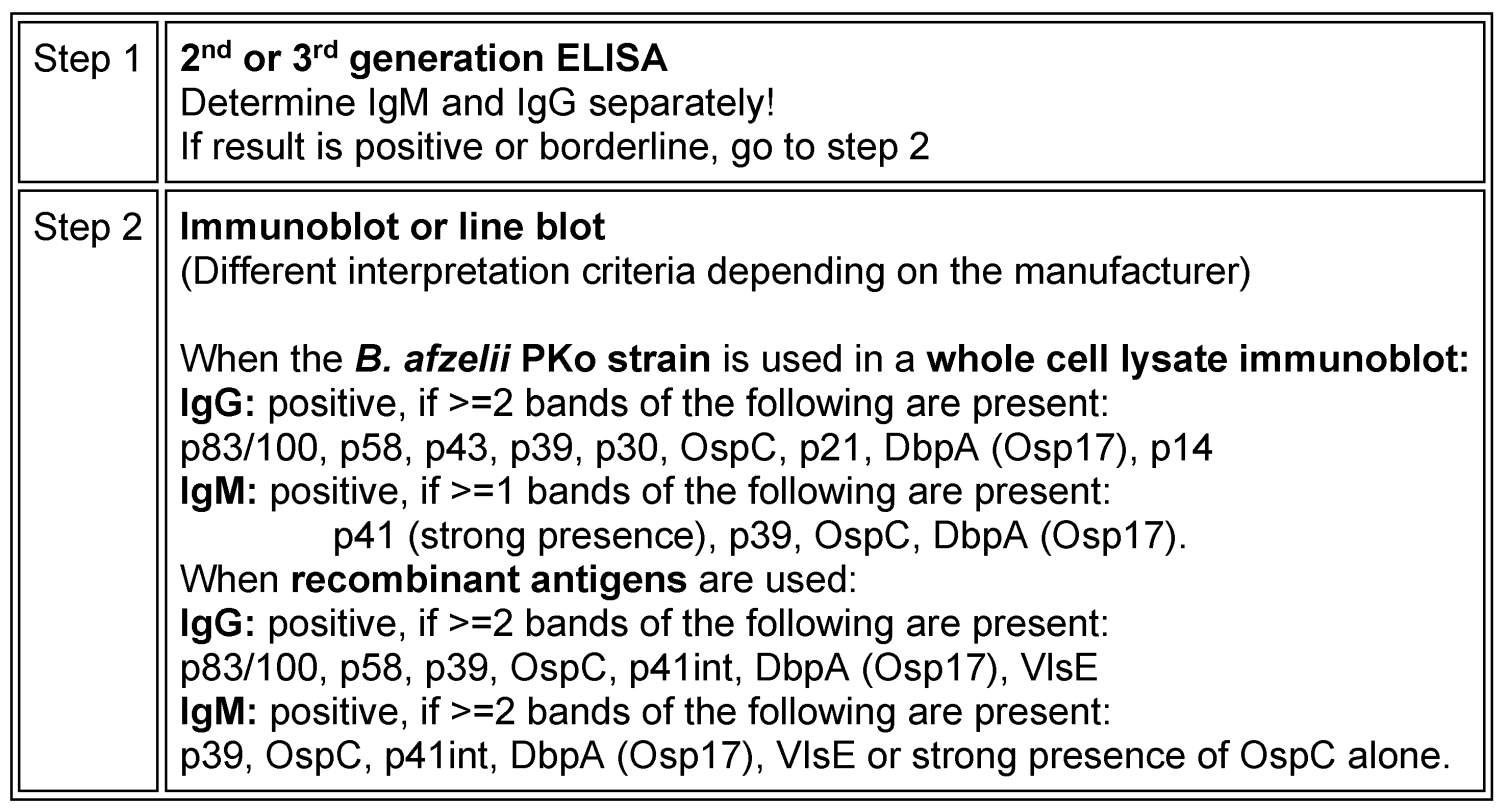
Two tiered serological diagnostic approach (as per MIQ 12 and DIN 58969-44 2005-07)

**Table 4:**
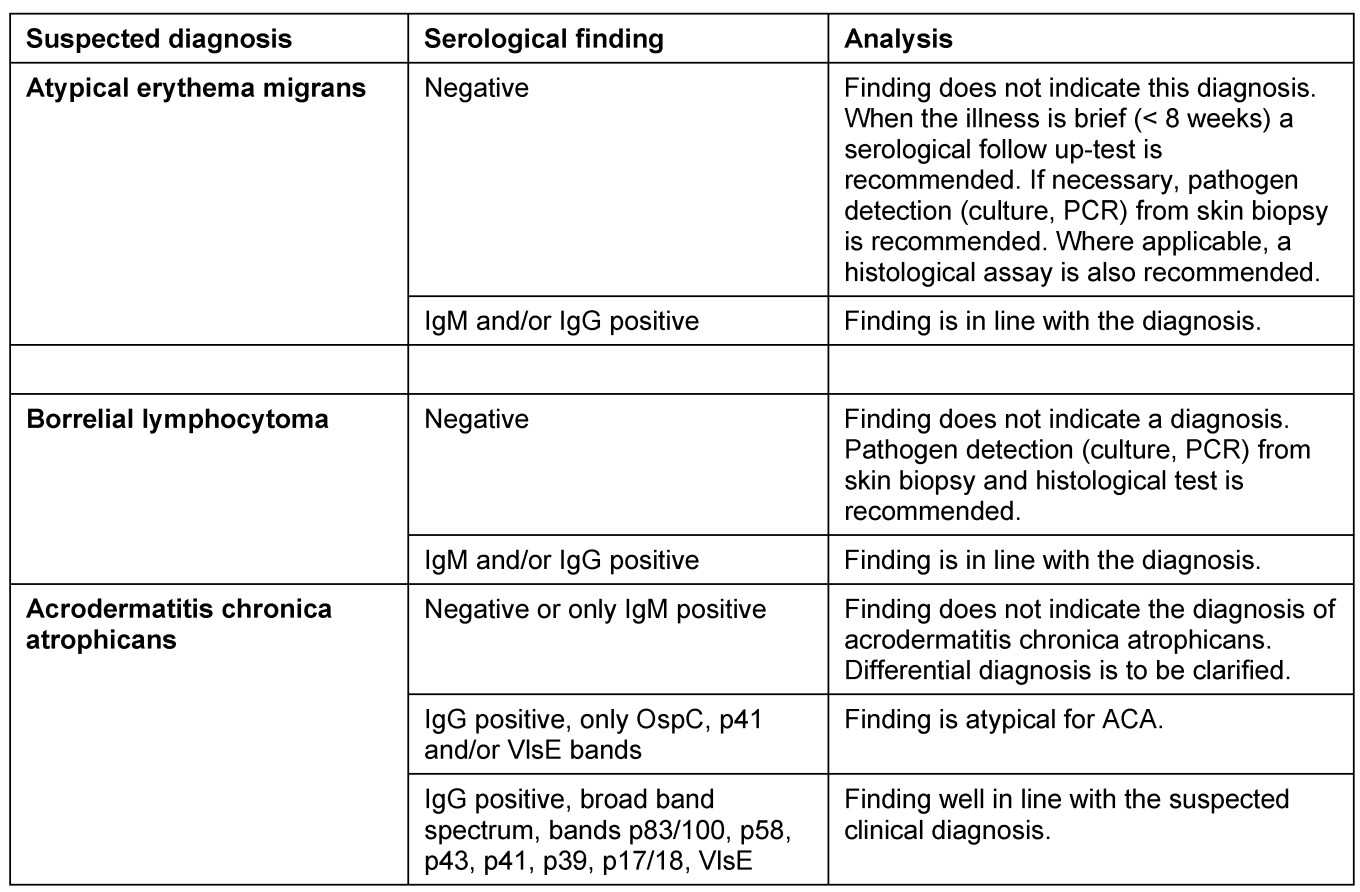
Interpretation of serological result constellations

**Table 5:**
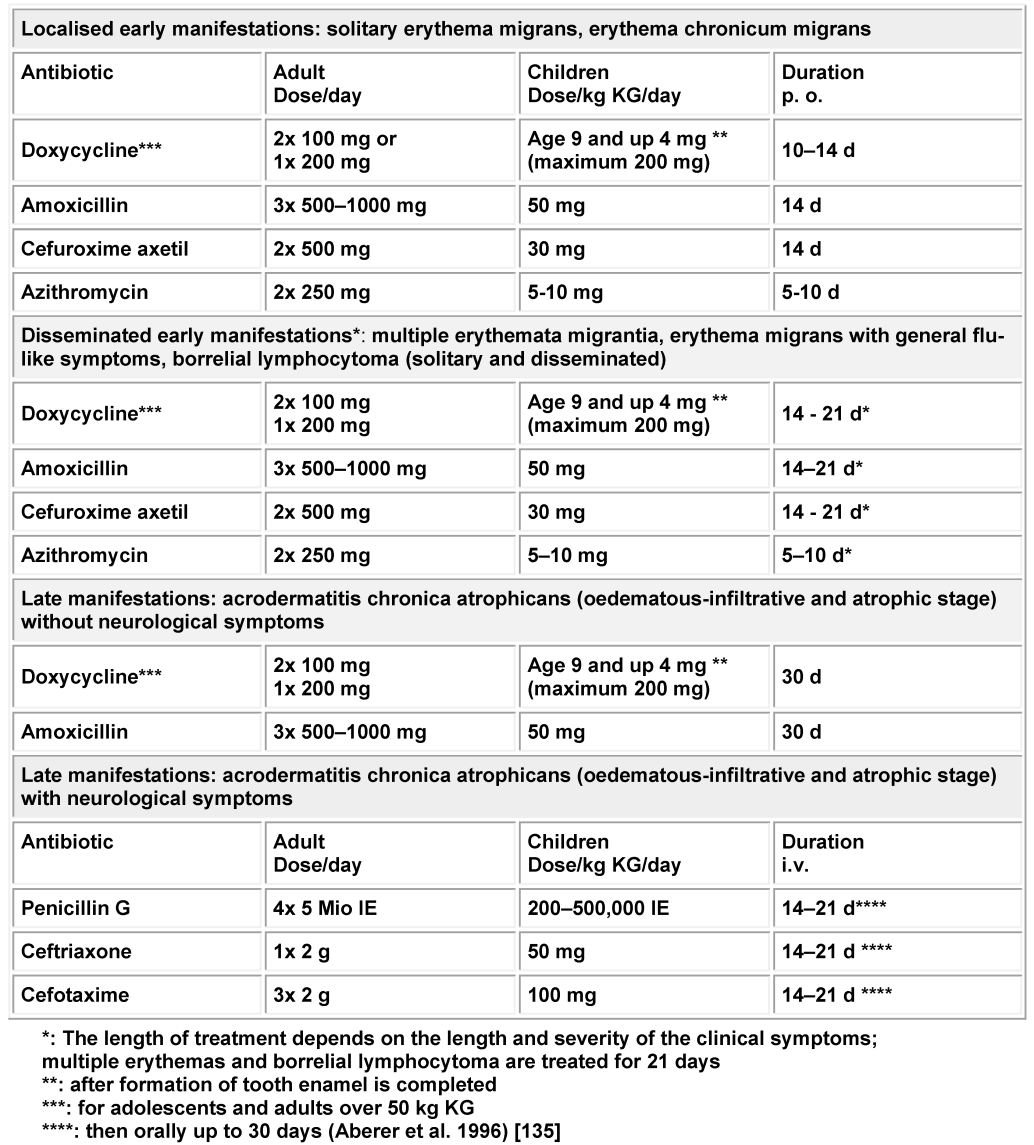
Treatment recommendations for cutaneous Lyme borreliosis
