# Supplementary material for: Cutaneous Lyme borreliosis: Guideline of the German Dermatology Society
Source: Ger Med Sci. 2017 Sep 5;15:Doc14. doi: 10.3205/000255 (PMC5588623; doi:10.3205/000255)
Supplement: Annex 1–4 [file GMS-15-14-s-001.pdf]

Attachment 1: Annex 1–4

Annex 1: Patient information on what to do in the event of a tick bite

1. **Remove the tick as quickly as possible.**
   Special tick tweezers or tick cards are the best way to do this.
   Pull or push the tick slowly and patiently out of the skin – without twisting or pre-treating it with oil or glue. Avoid squishing the body.
   If some of the suction organ remains in the skin (often misinterpreted as the "head"), you can remove it with a sterile needle or a curette, or have it removed by a physician. If the suction organ remains in the skin, there is no danger of the Borrelia being transferred.
2. **Carefully examine your body and especially the heads of children for more ticks.**
3. **Observe the skin near the site of the bite for 6 weeks.** A redness that appears directly after the bite as a result of the tick's saliva will disappear within several days. If the redness **reappears** or if the original redness enlarges to ≥5 cm, be sure to consult a doctor. This can be a sign of **erythema migrans (migrating rash)**, the early manifestation of Lyme disease.
4. **When there is a typical migrating rash near the site of the bite, it should be treated with antibiotics, preferably with doxycycline (for children aged 9 and up) or amoxicillin,** even when no blood test has been carried out or if no antibodies have been detected.
5. The dissemination of the Borrelia through the blood – even without a reddening of the skin – is noticeable by a flu-like feeling without respiratory symptoms. This may be a precursor to organ disease, e.g. of the joints or the nervous system. Consult a physician who will decide whether or not a blood screening for Borrelia antibodies is necessary.
6. Early-stage Lyme disease can be completely cured when antibiotic treatment is carried out in line with the guidelines. This also prevents late manifestations.
7. It is not necessary to examine the tick for Borrelia since a positive detection does not confirm whether the Borrelia were indeed transferred to the skin and whether, in the case of a transmission, this will lead to an infection. A negative result does not exclude the possibility of a transmission.
8. Only a small proportion of the people infected with Borrelia become ill! This is why a prophylactic treatment with oral antibiotics is not recommended.

Attachement 1 to:Hofmann H, Fingerle V, Hunfeld KP, Huppertz HI, Krause A, Rauer S, Ruf B, Consensus group. Cutaneous Lyme borreliosis: Guideline of the German Dermatology Society. GMS Ger Med Sci. 2017;15:Doc14.
DOI: 10.3205/000255

Annex 2: National and international guidelines on Lyme borreliosis

AWMF Guideline of the German Dermatology Society, Working Group for Dermatological Infectiology; Cutaneous Manifestations of Lyme Borreliosis
http://www.derma.de/fileadmin/derma/pdfs/ll_kutane_lyme_borreliose.pdf

AWMF Guideline of the German Society of Neurology DGN – Neuroborreliosis
http://www.awmf.org/uploads/tx_szleitlinien/030-071l_S1_Neuroborreliose_2012.pdf

Brouqui P, Bacellar F, Baranton G, Birtles RJ, Bjoërsdorff A, Blanco JR, Caruso G, Cinco M, Fournier PE, Francavilla E, Jensenius M, Kazar J, Laferl H, Lakos A, Lotric Furlan S, Maurin M, Oteo JA, Parola P, Perez-Eid C, Peter O, Postic D, Raoult D, Tellez A, Tselentis Y, Wilske B; ESCMID Study Group on Coxiella, Anaplasma, Rickettsia and Bartonella; European Network for Surveillance of Tick-Borne Diseases. Guidelines for the diagnosis of tick-borne bacterial diseases in Europe. Clin Microbiol Infect. 2004 Dec;10(12):1108-32. DOI: 10.1111/j.1469-0691.2004.01019.x [177]

Canadian Public Health Laboratory Network. The laboratory diagnosis of Lyme borreliosis: Guidelines from the Canadian Public Health Laboratory Network. Can J Infect Dis Med Microbiol. 2007 Mar;18(2):145-8. DOI: 10.1155/2007/495108 [178]

Deutsche Borreliosis Society 2011 Guidelines for the Diagnosis and Treatment of Lyme Borreliosis http://www.borreliose-gesellschaft.de/Texte/Leitlinien.pdf

Evison J, Aebi C, Francioli P, Péter O, Bassetti S, Gervaix A, Zimmerli S, Weber R. Borréliose de Lyme 2e partie: clinique et traitement [Lyme disease Part 2: clinic and treatment]. Rev Med Suisse. 2006 Apr;2(60):925-8, 930-4. [179]

Evison J, Aebi C, Francioli P, Péter O, Bassetti S, Gervaix A, Zimmerli S, Weber R. Borréliose de Lyme 1ère partie: épidémiologie et diagnostic [Lyme disease Part I: epidemiology and diagnosis]. Rev Med Suisse. 2006 Apr 5;2 (60):919-24.

Evison J, Aebi C, Francioli P, Péter O, Bassetti S, Gervaix A, Zimmerli S, Weber R. Borréliose de Lyme 3e partie: prévention, grossesse, états d'immunodéficience, syndrome post-borréliose de Lyme [Lyme disease Part 3: prevention, pregnancy, immunodeficient state, post-Lyme disease syndrome]. Rev Med Suisse. 2006 Apr;2(60):935-6, 938-40. [180]

Flisiak R, Pancewicz S; Polish Society of Epidemiology and Infectious Diseases. Diagnostyka i leczenie boreliozy z Lyme. Rekomendacje Polskiego Towarzystwa Epidemiologów i Lekarzy Chorób Zakaznych [Diagnostics and treatment of Lyme borreliosis. Recommendations of Polish Society of Epidemiology and Infectious Diseases]. Przegl Epidemiol. 2008;62(1):193-9. [181]

Huppertz HI, Christen HJ, Fingerle V, Heininger U. Lyme-Borreliose. In: Deutsche Gesellschaft für Pädiatrische Infektiologie, editor. DGPI-Handbuch Infektionen bei Kindern und Jugendlichen. 6., vollst. überarb. Aufl. Stuttgart: Thieme Verlag; 2013.

Ljøstad U, Mygland A. Lyme-borreliose hos voksne [Lyme borreliosis in adults]. Tidsskr Nor Laegeforen. 2008 May;128(10):1175-8. [182]

Mygland A, Ljøstad U, Fingerle V, Rupprecht T, Schmutzhard E, Steiner I; European Federation of Neurological Societies. EFNS guidelines on the diagnosis and management of European Lyme neuroborreliosis. Eur J Neurol. 2010 Jan;17(1):8-16, e1-4. DOI: 10.1111/j.1468-1331.2009.02862.x [183)

Société de pathologie infectieuse de langue française. 16e Conférence de consensus en thérapeutique anti-infectiese de la Spilf. Borréliose de Lyme: démarches diagnostiques, thérapeutiques et préventives. Texte long [Lyme borreliose: diagnostic, therapeutic and preventive approaches--long text]. Med Mal Infect. 2007 Dec;37 Suppl 3:S153-74. [184]

Wormser GP, Dattwyler RJ, Shapiro ED, Halperin JJ, Steere AC, Klempner MS, Krause PJ, Bakken JS, Strle F, Stanek G, Bockenstedt L, Fish D, Dumler JS, Nadelman RB. The clinical assessment, treatment, and prevention of lyme disease, human granulocytic anaplasmosis, and babesiosis: clinical practice guidelines by the Infectious Diseases Society of America. Clin Infect Dis. 2006 Nov;43(9):1089-134. DOI: 10.1086/508667 [160]

Richtlijn Lymeziekte 2013. Dutch Guideline
http://www.kiza.nl/sites/default/files/images/Richtlijn%20lymeziekte%20definitief%2018%20juli%202013.pdf

ILADS 2014
Cameron DJ, Johnson LB, Maloney EL. Evidence assessments and guideline recommendations in Lyme disease: the clinical management of known tick bites, erythema migrans rashes and persistent disease. Expert Rev Anti Infect Ther. 2014 Sep;12(9):1103-35. DOI: 10.1586/14787210.2014.940900 [185]

Annex 3: Overview of treatment recommendations for cutaneous Lyme borreliosis in international guidelines

**Guideline / treatment recommendation**	**Erythema migrans**	**Early disseminated form (without neurological manifestations)**	**Acrodermatitis chronica atrophicans (ACA)**
**USA**<br>IDSA<br>Wormser et al. 2006	Doxycycline 100 mg 2x day or<br>Amoxicillin 500 mg 3x day or<br>Cefuroxime axetil 500 mg 2x day<br>or<br>Clarithromycin 500 mg 2x day<br>or<br>Erythromycin 4 x 500 mg /kg<br><br>Duration 14–21 days<br>or<br>Azithromycin 500 mg/daily<br>Duration 7–10 days	Doxycycline 100 mg 2x day or<br>Amoxicillin 500 mg 3x day or<br>Cefuroxime axetil 500 mg 2x day<br><br>Duration 14–21 days	Doxycycline 100 mg 2x day or<br>Amoxicillin 500 mg 3x day or<br>Cefuroxime axetil 500 mg 2x day<br>Duration 14–28 days
**Germany**<br>**AWMF S1**<br>Hofmann et al. 2009	Doxycycline 100 mg 2x day or<br>Amoxicillin 500–1000 mg 3x day<br>or<br>Cefuroxime axetil 500 mg 2x day<br>Duration 14–21 days<br>or<br>Azithromycin 250 mg 2x day<br>Duration 5–10 days		Doxycycline 100 mg 2x day 28 days<br>or<br>Ceftriaxone 2g/day<br>or<br>Cefotaxime 2g 3x day<br>or<br>Penicillin G $20x\ 10^6$ U/day<br>Duration 14–21 days
**France**<br>Chidiac et al. 2006	Amoxicillin 500 mg 3x day or<br>Doxycycline 100 mg 2x day or<br>Cefuroxime axetil 500 mg 2x day<br>Duration 14–21 days<br>or<br>Azithromycin 500 mg/day<br>Duration 10 days	Doxycycline 200 mg/day<br>or<br>Amoxicillin 1g/d 3x day<br>or<br>Ceftriaxone 2g/day<br>Duration 14–21 days	Doxycycline 200 mg/day<br>Duration 28 days<br>or<br>Ceftriaxone 2g/day<br>Duration 14 days

Attachement 1 to:Hofmann H, Fingerle V, Hunfeld KP, Huppertz HI, Krause A, Rauer S, Ruf B, Consensus group. Cutaneous Lyme borreliosis: Guideline of the German Dermatology Society. GMS Ger Med Sci. 2017;15:Doc14. DOI: 10.3205/000255

Guideline / treatment recommendation	Erythema migrans	Early disseminated form (without neurological manifestations)	Acrodermatitis chronica atrophicans (ACA)
**Netherlands**<br>Steensma et al. 2004	Doxycycline 100 mg<br>2x day<br>Duration 10 days<br>or<br>Amoxicillin 500 mg<br>3x day<br>Duration 14 days<br>or<br>Azithromycin<br>500 mg/day<br>Duration 5 days	Doxycycline 100 mg<br>2x day<br>Duration 21 days<br>or<br>Ceftriaxone 2g/day<br>Duration 14 days	Doxycycline 100 mg<br>2x day<br>(30 days)
**Poland**<br>Flisiak and Pancewicz 2008	Amoxicillin 500 mg<br>3x day or<br>Doxycycline 100 mg<br>2x day or<br>Cefuroxime axetil<br>500 mg 2x day<br>Duration 14–21 days<br>or<br>Azithromycin<br>500 mg/day<br>Duration 7–10 days<br>or<br>Clarithromycin<br>500 mg 2x day<br>or<br>Penicillin V 1000 mg<br>3x day<br>Duration 14–21 days	-	Amoxicillin<br>500–1000 mg 3x day<br>or<br>Doxycycline 100 mg<br>2x day or<br>Ceftriaxone 2/day<br>or<br>Cefotaxime 2g 3x day<br>or<br>Penicillin G 3–4 x10^6 U<br>4x day<br>Duration 14–28 days
**Finland**<br>Oksi et al. 2008	Amoxicillin<br>500–1000 mg 3x day<br>or<br>Doxycycline 100 mg<br>2x day or<br>Cefuroxime axetil<br>500 mg 2x day<br>Duration 14 days<br>or<br>Azithromycin<br>500 mg/day<br>Duration 10 days	-	Ceftriaxone 2g/day<br>Duration 14–21 days<br>or<br>Amoxicillin 500 mg<br>3x day or<br>Doxycycline 100 mg<br>2x day<br>Duration 1–2 months

**Guideline / treatment recommendation**	**Erythema migrans**	**Early disseminated form (without neurological manifestations)**	**Acrodermatitis chronica atrophicans (ACA)**
**Norway**<br>Ljostad and Mygland 2009	Doxycycline 100 mg 2x day or 200 mg/day<br>or<br>Amoxicillin 500 mg 3x day<br>Duration 14 days	Doxycycline 100 mg 2x day or 200 mg/day<br>Duration 14 days	Doxycycline 200mg/day or 100mg 2x day<br>Duration 20 days
**Switzerland**<br>Evison et al. 2006	Doxycycline 100 mg 2x day<br>Duration10 days<br>or<br>Amoxicillin 500 mg 3x day<br>or<br>Cefuroxime axetil 500 mg 2x day or Clarithromycin 500 mg 2x day<br>Duration 14–21 days<br>or<br>Azithromycin 500 mg/day<br>Duration 7–10 days		-
2011 Guideline of the Germany Borreliosis Society	Doxycycline 400 mg/d<br>or<br>Amoxicillin 3000–6000 mg/d<br>or<br>Cefuroxime 2x 500 mg/d<br>or<br>Clarithromycin 500–1000 mg/d<br>or<br>Azithromycin 500 mg/ 3–4x/w<br>**Duration at least 4 weeks**	Ceftriaxone 2g/d iv<br>or<br>Cefotaxime 2–3x 4g/d iv<br>or<br>Minocycline 200mg/d p o<br>**Duration**<br>**Change antibiotic after 4 weeks if ineffective after clinical course**	

Guideline / treatment recommendation	Erythema migrans	Early disseminated form (without neurological manifestations)	Acrodermatitis chronica atrophicans (ACA)
**USA**<br>ILADS 2014	**Duration of application 10 to 21 days or duration of application 4–6 weeks (preference)**<br>Amoxicillin<br>1500–2000 mg/d<br>children: 50 mg/d distributed over 3 doses, max. 1500 mg<br>Cefuroxime<br>2x 500 mg/d<br>children: 20–30 mg/d distributed over 2 doses, max. 1000 mg<br>Doxycycline 2x 100 mg<br>children over 8: 4 mg/d distributed over 2 doses, max. 200 mg<br>Azithromycin 10 mg/kg on day 1, thereafter 5–10mg/kg/d<br>max. 500 mg/d<br>**If persists**: increase dosage as needed, possibly also combined with antibiotics that work intracellularly. Repeat the 4–6 week treatment if symptoms persist, like under Lyme arthritis, ACA, late-stage neuroborreliosis.		If symptoms recur, or progress after DD, repeat antibiotic treatment* with new alternative antibiotic** or combination therapy* possibly also i.m. penicillin G 1.2–3.6 units/week – or iv. Ceftriaxone 2g/d<br>- Tetracycline: 1000–1500 mg distributed over 3–4 doses<br>- (Cefotaxime)<br>**Note: ILADS stresses that currently **no** fixed treatment schemes have been established as a result of a lack of evidence. Duration and dosage depend on, among other things, severity of the illness and response to the treatment.
Recommendations of the Commission for Pharmacotherapy of the German Society of Rheumatology (DGRh)<br>Gaubitz M, et al.<br>Z Rheumatol 2014; 73: 469-474.	Doxycycline 100 mg<br>2x daily p.o.<br>or 200 mg 1xd daily p.o.<br>or<br>Amoxicillin 3 to 4 x 500–1000 mg<br>or<br>Cefuroxime 2x daily 500 mg p.o.<br>Duration 10–21 days<br>or<br>Azithromycin 2x 500 mg p.o. day 1, then 1x 500 mg p.o.<br>Duration 5 days		Doxycycline 100 mg<br>2x daily p.o.<br>or 200 mg 1xd daily p.o.<br>or<br>Amoxicillin 3 x 500–1000 mg<br>or<br>Ceftriaxone 1x daily 2 g i.v.<br>Duration 21 (14–30) days

Attachement 1 to:Hofmann H, Fingerle V, Hunfeld KP, Huppertz HI, Krause A, Rauer S, Ruf B, Consensus group. Cutaneous Lyme borreliosis: Guideline of the German Dermatology Society. GMS Ger Med Sci. 2017;15:Doc14. DOI: 10.3205/000255

Annex 4: Legal regulations

Reporting obligations

In Germany there are no general disease or pathogen-specific reporting obligations in accordance with the infection protection law (IfSG).

However, in the **states of Bavaria, Berlin, Brandenburg, Mecklenburg West Pomerania, Rhineland Palatinate, Saarland, Saxony, Saxony-Anhalt and Thuringia** there are additional ordinances that supplement the infection protection law. According to these state ordinances, the respective health departments must be informed when a person (unnamed) falls ill with or dies from Lyme borreliosis in the form of an erythema migrans, an acute case of neuroborreliosis or an acute case of Lyme arthritis.
http://www.rki.de/DE/Content/Infekt/EpidBull/Merkblaetter/Ratgeber_LymeBorreliose.html

Work-related illness

Reasonable suspicion that the Lyme borreliosis could be a work-related illness BK No. 3102 must be reported immediately to the accident insurance, e.g. the employer´s liability insurance association, by the attending physician in accordance with Article 202 of the Social Security Code VII (SGB). The category of this work-related illness is: diseases transmitted from animals to humans. As part of this process of determination, the accident insurance will order or carry out the necessary work-related and medical examinations. The professions most at risk include forestry workers, gardeners, agriculturalists and hunters [Triebig G, Kentner M, Schiele R, Hrsg. Arbeitsmedizin, 2. Auflage. Stuttgart: Gentner-Verlag; 2008.]

Attachement 1 to:Hofmann H, Fingerle V, Hunfeld KP, Huppertz HI, Krause A, Rauer S, Ruf B, Consensus group. Cutaneous Lyme borreliosis: Guideline of the German Dermatology Society. GMS Ger Med Sci. 2017;15:Doc14.
DOI: 10.3205/000255
